# Optimizing two-dose vaccine resource allocation to combat a pandemic in the context of limited supply: The case of COVID-19

**DOI:** 10.3389/fpubh.2023.1129183

**Published:** 2023-04-24

**Authors:** Jin Zhu, Qing Wang, Min Huang

**Affiliations:** College of Information Science and Engineering, Northeastern University, Shenyang, Liaoning, China

**Keywords:** vaccine allocation, COVID-19, epidemic modeling, nonlinear programming, pandemic control

## Abstract

The adequate vaccination is a promising solution to mitigate the enormous socio-economic costs of the ongoing COVID-19 pandemic and allow us to return to normal pre-pandemic activity patterns. However, the vaccine supply shortage will be inevitable during the early stage of the vaccine rollout. Public health authorities face a crucial challenge in allocating scarce vaccines to maximize the benefits of vaccination. In this paper, we study a multi-period two-dose vaccine allocation problem when the vaccine supply is highly limited. To address this problem, we constructed a novel age-structured compartmental model to capture COVID-19 transmission and formulated as a nonlinear programming (NLP) model to minimize the total number of deaths in the population. In the NLP model, we explicitly take into account the two-dose vaccination procedure and several important epidemiologic features of COVID-19, such as pre-symptomatic and asymptomatic transmission, as well as group heterogeneity in susceptibility, symptom rates, severity, etc. We validated the applicability of the proposed model using a real case of the 2021 COVID-19 vaccination campaign in the Midlands of England. We conducted comparative studies to demonstrate the superiority of our method. Our numerical results show that prioritizing the allocation of vaccine resources to older age groups is a robust strategy to prevent more subsequent deaths. In addition, we show that releasing more vaccine doses for first-dose recipients could lead to a greater vaccination benefit than holding back second doses. We also find that it is necessary to maintain appropriate non-pharmaceutical interventions (NPIs) during the vaccination rollout, especially in low-resource settings. Furthermore, our analysis indicates that starting vaccination as soon as possible is able to markedly alleviate the epidemic impact when the vaccine resources are limited but are currently available. Our model provides an effective tool to assist policymakers in developing adaptive COVID-19 likewise vaccination strategies for better preparedness against future pandemic threats.

## 1. Introduction

Infectious disease, in particular viral infections, poses a significant public health threat and socio-economic confounding. Currently, an immediate example is the ongoing global coronavirus disease 2019 pandemic caused by severe acute respiratory syndrome coronavirus 2 (SARS-CoV-2), which continues to spread worldwide and ultimately affected the lives of hundreds of millions of people since December 2019. In view of the disease entering into a global exponential growth phase, the COVID-19 outbreak was declared a global pandemic by World Health Organization (WHO) on March 11, 2020 (1).

To combat the COVID-19 pandemic, a range of stringent non-pharmaceutical interventions (NPIs), such as national or state-wide lockdowns, travel restrictions, prohibitions on mass gatherings, and maintaining secure social distancing, has been introduced in many countries (2). These intervention strategies are able to significantly decrease the frequency of contact between infected and susceptible populations and have been shown to be effective measures to slow down the propagation speed of the COVID-19 pandemic (3). However, NPIs against COVID-19 are not a sustainable solution in the long term because of the substantial economic cost and result in a negative impact on normal social activities (4). According to the International Monetary Fund (IMF) reports, the great lockdown to contain the COVID-19 pandemic has triggered the worst economic downturn since the Great Depression (5).

The adequate vaccination provides a promising and sustainable long-term solution to mitigate the spread of COVID-19. Several safe and effective COVID-19 vaccines have been developed, tested, and approved at an unprecedented pace, driven by the joint efforts of drug researchers around the world after the outbreak (6). Unfortunately, however, a supply shortage is inevitable during the early stage of any new vaccine rollout as the limited production and distribution capacity (7). Decision makers face a crucial public health conundrum of how to allocate scarce vaccines in resource-constrained settings to maximize the benefits of vaccination (8).

Determining the optimal vaccine allocation strategy during the COVID-19 pandemic is a challenging problem. On the one hand, the spread of COVID-19 is a highly complex process. In contrast to the previous coronavirus virus, COVID-19 exhibits several peculiar epidemiological features. For example, some studies have demonstrated the substantial contribution of pre-symptomatic and asymptomatic infections to COVID-19 transmission (9, 10). Public health authorities need a better understanding of such inapparent transmission occurring *via* person-to-person interaction will play major roles in the prevention and control the COVID-19. In addition, COVID-19 transmission can show noticeable group differences. In other words, several risk factors within the population may have contributed to the heterogeneous transmission of COVID-19, such as children and adolescents have been shown to have lower susceptibility to infection compared with elders (11, 12), older adults have a greater risk of becoming symptomatic than younger (13, 14), beyond this, the risk of requiring hospitalization and risk of death also displayed a similar characteristic (15–17). On the other hand, most of the currently available vaccines require two doses given in tandem over a certain time interval (18–20). A two-dose vaccination schedule faces more complicated and realistic logistics challenges than a simple single-dose vaccination rollout. Specifically, in the context of limited vaccine supply, it is necessary to determine not only the optimal allocation strategies with the first and second doses of vaccine in each time period but also to ascertain if reserving the doses for individuals who have received the first dose to avoid the failure of their first dose vaccine due to insufficient supply. Hence, the two-dose vaccination procedure should not be ignored during the COVID-19 vaccine resources allocation process such that all the available vaccine resources can be administered to the people who need them the most (21).

To address the above-mentioned challenges, in this paper, we investigate a multi-period two-dose vaccine resource allocation problem to deal with a pandemic when the vaccine supply is highly limited. First, we constructed a novel age-structured compartmental model tailored to COVID-19 transmission. More specifically, we modified a deterministic susceptible-exposed-infected-recovered (SEIR) compartmental model by introducing the two-dose vaccination procedure and adding additional compartments to capture different states of the virus propagation. In our model, the different infected individuals are distinguished based on whether they presented with symptoms and on the severity of symptoms. In addition to this, our model also takes into account group heterogeneity, encompassing the group difference in terms of susceptibility, symptom rates, severity, etc. Then, we propose a nonlinear programming (NLP) model that integrates the proposed compartmental model to allocate scarce vaccines resource for suppressing the negative effects caused by the virus transmission. The objective of this model is to minimize the total number of potential deaths in the population over a multi-period planning time horizon. Afterward, we conducted a real-world case study regarding the 2021 COVID-19 vaccination campaign in the Midlands of England to verify the performance of the proposed model. Finally, we also give some practical recommendations for a two-dose vaccine allocation strategy in the context of the COVID-19 pandemic.

The main contribution of this paper is as follows: (i) We present an age-structured SEIR-type compartment model to describe the course of COVID-19 transmission, which explicitly considers the two-dose vaccination procedure and several important epidemiologic features of COVID-19, such as pre-symptomatic and asymptomatic transmission, group heterogeneity in susceptibility, symptom rates, severity, as well as multiple mechanisms of vaccine action, etc. (ii) We propose a multi-period two-dose vaccine allocation problem to assist public health authorities in making optimal allocation decisions for COVID-19 pandemic control. To this end, a nonlinear programming model is formulated to minimize the total number of deaths under a limited supply of vaccine resources. (iii) We performed a retrospective study based on a real-world COVID-19 vaccination campaign, which aims to provide some important insights into the strategies for allocating scarce vaccine resources.

The rest of this paper is organized as follows. In Section 2, we review the relevant literature. In Section 3, we develop a multi-period vaccine allocation optimal model that integrates a compartmental model tailored to COVID-19 transmission. In Section 4, we elaborate on a case study based on a real-world COVID-19 outbreak to illustrate the performance of the proposed model. The key results and discussion are presented in Section 5, and finally, conclusions and suggestions for future research are summarized in Section 6.

## 2. Literature review

Infectious disease modeling plays a crucial role in understanding the evolution of infectious diseases and planning for public health responses to mitigate an infectious disease outbreak (22). Typically, the compartmental model provides a powerful tool to describe infectious disease transmission dynamics. One of the most common compartmental models was called the Susceptible-Infectious-Removed (SIR) model, which was first proposed by Kermack and Mckendrick (23). The authors classify the population into three mutually exclusive compartments according to its infection status, namely susceptible, infected, and recovered. Individuals in each compartment are assumed to mix homogeneously, and the transition among these compartments can be described by a system of nonlinear differential equations. Despite its structural simplicity, the SIR model is exceedingly useful and able to easily be extended based on the transmission characteristics of different pathogens. For example, the well-known SEIR model, which considers the incubation period by means of introducing a compartment for exposed individuals, has drawn considerable attention (24). After that, the SEIR model and many of its variants were widely applied in studying the transmission of specific infectious diseases such as SARS (25–27), H1N1 (28–30), Smallpox (31–33), Ebola (34, 35), and Cholera (36, 37).

Currently, the classical SEIR model has also been used to model the recent outbreak of COVID-19 (38–40). Furthermore, several more sophisticated models have been developed to incorporate more important epidemiologic features that can more accurately depict the transmission of the COVID-19 pandemic. For example, Hao et al. (41), Aleta et al. (42), and Whittaker et al. (43) extend the standard SEIR model by considering the different categories of infectious individuals to simulate the transmission dynamics of COVID-19. These studies classified infected according to infectious health state, including pre-symptomatic, asymptomatic, and symptomatic individuals. Walker et al. (44) constructed an age-structured SEIR model, which explicitly takes into account disease severity and healthcare levels to explore the spread of COVID-19 under different health capacity conditions. Viana et al. (45) proposed an age-structured SEIR model including vaccination to evaluate the different scenarios for the relaxation of social distancing measures during the vaccination rollout. Yang et al. (46) developed an SEIR-type model incorporating hospitalized in the general ward and hospitalized in the intensive care unit (ICU), aiming to derive optimal switching strategies between different community mitigation stages based on the daily number of admissions. In the studies above, such compartmental models are generally applied to simulate the spread of infectious diseases in human populations, and these studies focus on evaluating the effectiveness of various non-pharmacological intervention strategies to control the epidemic.

Pharmacologic interventions to deal with an outbreak are another important area of research. In particular, the vaccination campaign has provided a long-term solution, which is regarded as a powerful tool for suppressing infectious disease pathogen transmission (47). Notably, vaccine availability will usually be insufficient when faced with a previously unknown pathogen, especially in the early stage of vaccine rollout (48). The significant challenge facing public health authorities is how to allocate scarce vaccines to mitigate the negative effects of a pandemic. This type of problem is referred to as vaccine allocation for epidemic control, and it has recently received widespread attention from the scientific community attention (37, 47, 49–56).

Several studies have considered using the mathematical programming approach that incorporates compartmental models to address the vaccine allocation optimization problem for epidemic control. For instance, Ren et al. (57) studied vaccine allocation strategy during a smallpox outbreak, where infectious disease spread is described by an SIR compartment model. The authors came up with an approximate method for the representation of the disease dynamics, which aims to formulate this allocation problem as a mixed integer programming model. Furthermore, they also proposed an efficient heuristic algorithm to solve large-scale problems in a reasonable time. Duijzer et al. (58) used a standard SIR model to describe the course of infectious disease transmission in a heterogeneous population. Subsequently, they developed a nonlinear programming model that minimizes the number of vaccine doses used under the effective reproductive number equal to one. The authors provided an efficient solution method based on Perron–Frobenius theory to find the optimal vaccine allocation. In a similar study, Enayati and Ozaltin (53) used the well-known Gini coefficient to determine a balanced influenza vaccine allocation strategy as regards efficiency and equity. They find that group-specific transmission is important in the evolution of the influenza virus and should be taken into account in vaccine allocation decision-making. Ng et al. (59) combined a multi-criterion mathematical programming model with an SIR model to determine the optimal vaccination strategies for seasonal influenza. The proposed multi-criterion optimization problem was solved by the augmented epsilon-constraint method. They showed that the group-targeted vaccination strategy outperforms both the mass and random vaccination strategies. A number of recent studies focused on optimal vaccine allocation strategies for COVID-19 based on age-structured compartmental models. Miura et al. (55) applied an age-structured SIR model to simulate the COVID-19 epidemic trajectories and present a data-driven approach for vaccine allocation. The authors find that optimal vaccine allocation strategies depend on the objective of epidemic control. A similar conclusion has been reached in the work of Matrajt et al. (54) and Molla et al. (60). In another study, Jarumaneeroj et al. (47) proposed an age-structured SIQRV (Susceptible-Infectious-Quarantined-Recovered-Vaccinated) model to delineate the transmission dynamics of COVID-19 and later formulated a nonlinear programming model to obtain the optimal vaccine allocation strategies with the aim to minimize the total weighted burden on the health care system over a multi-period planning horizon. More recently, Tetteh et al. (61) studied the mass and ring vaccination strategies with different vaccine efficacy and population coverage using stochastic network models. González-Parra et al. (62) proposed two nonlinear mathematical models and applied them to explore the optimal vaccine allocation strategy under different scenarios. While these studies have provided valuable management insights regarding the vaccine allocation decisions in the context of an outbreak, they rely on the simplified assumption that the course of vaccination contains only single-dose. However, in the case of some infectious diseases (e.g., COVID-19, Cholera), most approved vaccines require two doses were administered at certain time intervals (6, 37). Obviously, the two-doses vaccination procedure is more complex than the single-dose vaccination, which is often ignored in previous studies on the vaccine allocation optimization problem.

The two-dose vaccine allocation problem to combat a pandemic in the context of limited capacity has received less attention in the literature. Among the few studies on this issue, Matrajt et al. (54) and Leung et al. (37) used mathematical models combined with optimization algorithms to determine the optimal allocation strategy with one and two doses of vaccine under various outbreak settings with different combinations of parameters. There are also some research efforts that have attempted to evaluate the effectiveness of delaying the second dose strategy with the COVID-19 vaccine (6, 63, 64). These studies assume immune protection immediately following vaccination. However, a number of studies have shown that there is a delay between injecting a dose and the onset of dose-specific protection (17, 65), which should not be ignored in the mathematical modeling of vaccination in response to an outbreak.

The most relevant study to our work was conducted by Parino et al. (21). They proposed a nonlinear programming model to optimize a multi-period two-dose vaccine allocation problem during the COVID-19 outbreak. Our work differs from Parino et al. (21) in three main aspects. First, we incorporate the age-structured population in the multi-period two-dose vaccine allocation decision-making process because age is regarded as a significant risk factor for the transmission of COVID-19. Specifically, the study not only allows us to determine the amount of dose used for the first and second vaccination, respectively, but also provides more detailed strategies for two-dose vaccine allocation by age. Second, the explicit modeling of inapparent transmission from pre-symptomatic and asymptomatic infections in our model and the separation of distinct levels of transmissibility for different types of infections. Third, we consider the specific features of the COVID-19 vaccine, including the delay effect between vaccination with a dose and the onset of dose-specific immune responses, and the current vaccine provides multiple mechanisms of action (for example, by vaccination reduction in risk for infection, reduction in risk for developing symptoms after infection, or reduction in risk for severe symptoms).

## 3. Pandemic transmission and vaccine allocation model

**Figure 1:**
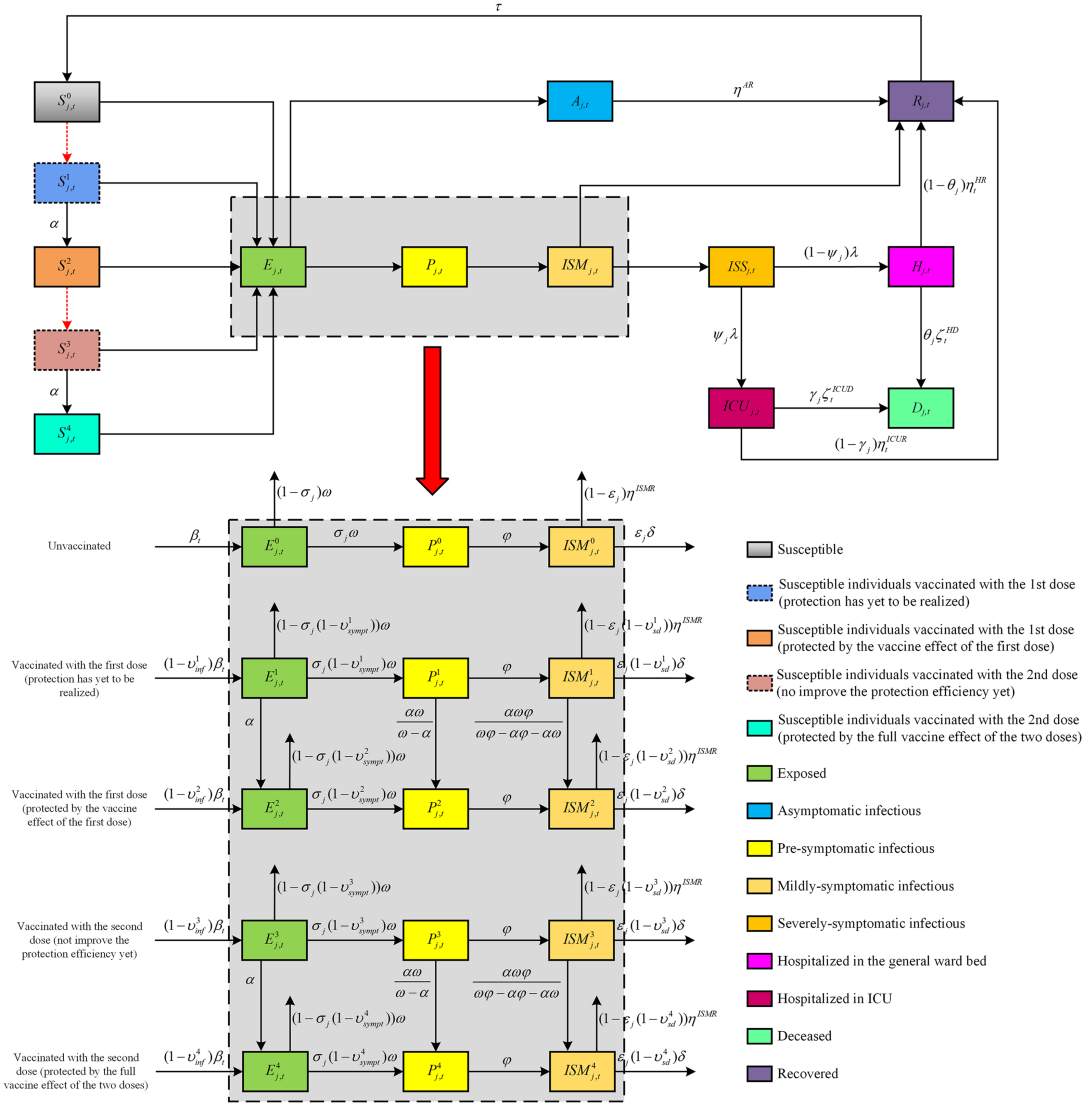
Schematic representation of the epidemiological model and transitions between compartments.

In this section, the details of the proposed mathematical model are introduced to delineate the course of disease progression and the vaccination process, and we present a mathematical programming formulation of the two-dose vaccine allocation problem. As shown in Figure 1, the proposed model describes the transmission of the viral pathogens as an evolving dynamic among different epidemiological states in each population group stratified according to age and vaccination status for each time period, based on an extension of the discrete-time, age-structured, deterministic SEIR-type compartmental model. Specifically, the total population is partitioned into 16 5-year age groups (from 0 to 79 years with 5-year increments) and one age group over 80 years. Each age group is further divided into five subgroups account for vaccination status: (i) unvaccinated individuals; (ii) individuals vaccinated with the first dose (protection has yet to be realized); (iii) individuals vaccinated with the first dose (protected by the vaccine effect of the first dose); (iv) individuals vaccinated with the second dose (not improve the protection efficiency yet); (v) individuals vaccinated with the second dose (protected by the full vaccine effect of the two doses). For each population class, we tracked ten infection statuses: susceptible (*S*), exposed (*E*), asymptomatic infectious (*A*), pre-symptomatic infectious (*P*), mildly-symptomatic infectious (*ISM*), severely-symptomatic infectious (*ISS*), hospitalized in the general ward bed (*H*), hospitalized in the ICU (*ICU*), recovered (*R*) and deceased (*D*). In this figure, the compartments correspond to the infection and vaccination status of individuals in a population affected by the pandemic, and the arrows correspond to the flow between different statuses, while the rates of transition are marked next to the arrows. For simplicity of visualization, we only display the vaccination status transition between epidemiological compartments for susceptible, exposed, pre-symptomatic infectious, and mildly-symptomatic infectious. Apparently, the change of the individual status either depends on disease/clinical (black solid arrows) or vaccine allocation decision-making (red dashed arrows).

With the pathogen continuing to transmit within a population, the individual is initially susceptible, who becomes exposed (but not yet infectious) through human-to-human direct contact in the community with infectious individuals. After the latent period, they will either become pre-symptomatic or asymptomatic individuals. After this, those who are asymptomatic individuals will recover naturally without any intervention or hospitalization. Additionally, the pre-symptomatic individuals progress to mildly-symptomatic infectious. They will either transition to recovery by natural healing in the same manner as asymptomatic individuals, or the remaining fraction will become severely-symptomatic infectious requiring hospitalization. Of these, a proportion of severely-symptomatic infectious developed hospitalized in the general ward bed, while the remainder of severely-symptomatic infectious was hospitalized in the ICU because they required ventilator support or ICU care. Finally, the hospitalized individual will receive medical treatment and may recover completely, while they may also deteriorate and subsequently die. Notably, recovered individuals will wane of infection-derived temporary immunity, after which they return to being fully susceptible.

On the other hand, the vaccine allocation decision-making concerns different vaccination statuses of individuals. If available vaccine capacities exist, these can either be used to administer the first dose or second dose of the vaccine. Moreover, since the vaccine protection is not effective immediately, there is a delay between administration (both first and second dose) and the onset of dose-specific immune responses (60). It is to be mentioned that a minimum/maximum vaccination time interval exists between the first and second doses in order to prevent vaccine failure (64). In this research, we consider a leaky vaccine that partially reduces the risk of infection, developing symptoms after infection, and severe symptoms. To facilitate the modeling and interpretation, we summarize the notations used throughout the rest of this paper in Section 3.1.

### 3.1. Model notations

A summary of the model notations and their description is presented in the following.

#### 3.1.1. Sets

*T*Set of time periods.

*J*Set of age groups.

*K*Set of vaccination statuses.

#### 3.1.2. Indices

*t*Index for time period where t∈T .

$j,j^{\prime }$ Index for age group where $j,j^{\prime }\in J$ .

*k*Index for vaccination status where k∈K .

#### 3.1.3. Epidemiological parameters

$\beta_{t}$ Time-varying transmission coefficient.

$c_{j,j^{\prime }}$ Contact rate between age group *j* and group $j^{\prime }$ .

$\rho_{j}$ Relative susceptibility to infection of age group *j.*

$\chi^{A}$ Relative infectiousness of asymptomatic infectious individuals.

$\chi^{P}$ Relative infectiousness of pre-symptomatic infectious individuals.

$\upsilon_{\inf}^{k}$ Vaccine efficacy against infection in vaccination status *k.*

1α Mean duration of delay between receiving vaccine injection and the onset of dose-specific effectiveness.

1τ Mean duration of naturally acquired immunity.

1ω Mean latent period.

$\sigma_{j}$ Proportion of exposed individuals of age group j who become pre-symptomatic.

$\upsilon_{\mathrm{sympt}}^{k}$ Vaccine efficacy against symptomatic disease in vaccination status *k.*

1φ Mean duration of the pre-symptomatic infectious period.

1δ Mean duration of the mildly-symptomatic infectious period.

$\varepsilon_{j}$ Proportion of mildly-symptomatic infectious individuals of age group *j* who develop severe disease requiring hospitalization.

$\upsilon_{sd}^{k}$ Vaccine efficacy against severe disease in vaccination status *k.*

$\psi_{j}$ Proportion of hospitalized cases of age group *j* who required ICU.

1λ Mean duration of severe infection prior to hospitalization.

$\gamma_{j}$ Proportion of ICU cases of age group *j* who died.

$\theta_{j}$ Proportion of non-ICU cases of age group *j* who died.

1ηAR Mean recovery time of asymptomatic infectious individuals.

1ηISMR Mean recovery time of mildly-symptomatic infectious individuals.

1ηtICUR Mean recovery time of ICU cases at time *t.*

1ηtHR Mean recovery time of non-ICU cases at time *t.*

1ξtICUD Mean time of ICU cases who died at time *t.*

1ξtHD Mean time of non-ICU cases who die at time *t.*

1ςintervalU Maximum time interval between the administration of the first dose and second dose.

1ςintervalL Minimum time interval between the administration of the first dose and second dose.

#### 3.1.4. Other parameters

$B_{t}$ Number of vaccine doses supplied at time period *t.*

$C_{t}$ Total available vaccine capacities at time period *t.*

$IC_{j}^{*}$ Initial condition of the variable * of age group *j*.

$N_{j}$ Population size of age group *j.*

#### 3.1.5. Epidemiological variables

$S_{j,t}^{0}$ Number of unvaccinated susceptible individuals in age group *j* at time *t.*

$S_{j,t}^{k}$ Number of susceptible individuals in age group *j* and vaccination status *k* at time *t.*

$E_{j,t}$ Number of exposed individuals in age group *j* at time *t.*

$E_{j,t}^{0}$ Number of unvaccinated exposed individuals in age group *j* at time *t.*

$E_{j,t}^{k}$ Number of exposed individuals in age group *j* and vaccination status *k* at time *t.*

$A_{j,t}$ Number of asymptomatic infectious individuals in age group *j* at time *t.*

$P_{j,t}$ Number of pre-symptomatic infectious individuals in age group *j* at time *t.*

$P_{j,t}^{0}$ Number of unvaccinated pre-symptomatic infectious individuals in age group *j* at time *t.*

$P_{j,t}^{k}$ Number of pre-symptomatic infectious individuals in age group *j* and vaccination status *k* at time *t.*

$\mathrm{IS}M_{j,t}$ Number of mildly-symptomatic infectious individuals in age group *j* at time *t.*

$\mathrm{IS}M_{j,t}^{0}$ Number of unvaccinated mildly-symptomatic infectious individuals in age group *j* at time *t.*

$\mathrm{IS}M_{j,t}^{k}$ Number of mildly-symptomatic infectious individuals in age group *j* and vaccination status *k* at time *t.*

$\mathrm{IS}S_{j,t}$ Number of severely-symptomatic infectious individuals in age group *j* at time *t.*

$ICU_{j,t}$ Number of infected-hospitalized cases in the ICU for age group *j* at time *t.*

$H_{j,t}$ Number of infected-hospitalized cases in the general ward for age group *j* at time *t.*

$R_{j,t}$ Number of recovered individuals in age group *j* at time *t.*

$D_{j,t}$ Number of deceased individuals in age group *j* at time *t.*

#### 3.1.6. Decision variables

$x_{j,t}$ Number of vaccines allocated to individuals of age group *j* who administer the first dose at time period *t*.

$y_{j,t}$ Number of vaccines allocated to individuals of age group *j* who administer the second dose at time period *t*.

### 3.2. Model assumptions

The vaccine allocation model is constructed based on the complex transmission mechanisms of COVID-19. For simplicity, we make the following assumptions. First, in our model, we assume that individuals mix homogeneously within each compartment. We did not consider births and natural deaths in the population because the human lifespan is substantially longer than the duration of the outbreak (13). Furthermore, we also did not take into account population mobility in the modeling. In other words, the total population size remains constant, which has been deemed to be reasonable to consider for a short time frame (47, 66). We assumed that all severely-symptomatic (not hospitalized) individuals self-isolated and did not transmit the infection to others, as proposed in the work of Moghadas et al. (63). In addition, we assumed that once hospitalized, strict precautions were taken in hospitals so that individuals were no longer infectious (54, 67). Similar to Hogan et al. (68), we also make the simplifying assumption that all mortalities take place in the hospital. Second, although multiple COVID-19 vaccines have been authorized for human use, we assume that all types of vaccines have the same efficiency in our model (60, 69). Only susceptible individuals get the vaccination, a similar assumption in several studies, see, e.g., (67) and (16). Moreover, we also assume that the vaccinated infected individuals have the same transmission rate as those who are unvaccinated infected individuals (70). Finally, the model takes into account a continuous relaxation of the epidemiological variables and decision variables for efficiency and simplicity (21). However, this is typically assumed in epidemiological research models, and which implements this relaxation was confirmed to be effective enough to ensure high-quality results (66).

### 3.3. Mathematical formulation and description of the optimization model for vaccine allocation

Using the notations and assumptions mentioned above, we formulate the multi-period two-dose vaccine allocation model as follows.

#### 3.3.1. Objective function

The objective function of the proposed model is to minimize the total number of deaths, as can be seen in Equation (1), which includes the cases who died in the ICU (the first additive term) and died in the general ward (the second additive term) across age groups over the planning time horizon.

(1) $$\min\underset{t\in T}{\sum }\underset{j\in J}{\sum }\left(\gamma_{j}\zeta_{t}^{\mathrm{ICUD}}ICU_{j,t}+\theta_{j}\zeta_{t}^{HD}H_{j,t}\right)\text{.}$$

#### 3.3.2. Pandemic transmission dynamics constraints

In this subsection, we formulate the constraints related to pandemic transmission dynamics in Equations (2)–(12) to describe the movement of individuals between the disease states, which are shown in Figure 1.

##### 3.3.2.1. Initial conditions

We introduced a set of constraints (see Equation 2) to define the initial conditions for the number of individuals in each disease state in each age group at the beginning of the planning horizon. It is noteworthy that, due to the vaccination campaign not yet started, the initial condition for the disease states correlated with vaccination is zeros.

(2) $$\begin{array}{l} S_{j,t}^{0}=IC_{j}^{S^{0}},E_{j,t}^{0}=IC_{j}^{E^{0}},A_{j,t}=IC_{j}^{A},P_{j,t}^{0}=IC_{j}^{P^{0}},\mathrm{IS}M_{j,t}^{0}=IC_{j}^{\mathrm{IS}M^{0}},\\ \mathrm{IS}S_{j,t}=IC_{j}^{ISS},ICU_{j,t},=IC_{j}^{ICU},H_{j,t}=IC_{j}^{H},R_{j,t}=IC_{j}^{R},D_{j,t}=IC_{j}^{D},\\ \forall j\in J,t=1. \end{array}$$

##### 3.3.2.2. Susceptible individuals

Equation (3a) defines the number of unvaccinated susceptible individuals in age group *j* at the end of period *t* + 1 to be equal to the number of unvaccinated susceptible individuals of age group *j* in the previous period, plus the number of recovered individuals in age group *j* who transition to the fully susceptible state owing to the waning of infection-derived temporary immunity at the time period *t*, minus the number of unvaccinated susceptible individuals in age group *j* who transition to the exposed state due to contact with pathogens infection at the time period *t*, minus the number of unvaccinated susceptible individuals in age group *j* who receive the first dose at the time period *t*. In this equation, the term


$$\rho_{j}\beta_{t}\underset{j'\in J}{\sum }\frac{c_{j,j'}\left(\chi^{A}A_{j',t}+\chi^{P}P_{j',t}+\mathrm{IS}M_{j',t}\right)}{N_{j'}}\text{,}$$

represents the force of infection for age group *j*, where $\rho_{j}$ is the age-specific susceptibility of individuals to infection. The parameter $\beta_{t}$ denotes the time-varying transmission coefficient, and the parameters $\chi^{A}$ and $\chi^{B}$ represent the relative infectiousness of asymptomatic infectious and pre-symptomatic infectious individuals as compared to mildly-symptomatic infectious individual transmissions. Here we use $c_{j,j^{\prime }}$ to capture the person-to-person contact rate between individuals in age groups j and $j^{\prime }$ .


(3a) $$\begin{array}{l} S_{j,t+1}^{0}=S_{j,t}^{0}+\tau R_{j,t}-\rho_{j}\beta_{t}S_{j,t}^{0}\\ \underset{j'\in J}{\sum }\frac{c_{j,j'}\left(\chi^{A}A_{j',t}+\chi^{P}P_{j',t}+\mathrm{IS}M_{j',t}\right)}{N_{j'}}-x_{j,t},\;\forall j\in J,t\in T\text{.} \end{array}$$

The variation in the number of susceptible individuals vaccinated with the first dose (protection has yet to be realized), susceptible individuals vaccinated with the first dose (protected by the vaccine effect of the first dose), susceptible individuals vaccinated with the second dose (no improve the protection efficiency yet), and susceptible individuals vaccinated with the second dose (protected by the full vaccine effect of the two doses) were modeled separately in Equations (3b)–(3e) follow a similar logic, where 1/α is a delay between receiving vaccine injection and the onset of dose-specific effectiveness. It is noteworthy that in our model, we consider a leaky vaccine that partially reduces the risk of infection. We use $\upsilon_{\inf}^{k}$ to represent the vaccine effectiveness against infection in the vaccination status *k*.


(3b) $$\begin{array}{l} S_{j,t+1}^{1}=S_{j,t}^{1}+x_{j,t}-\left(1-\upsilon_{\inf}^{1}\right)\rho_{j}\beta_{t}S_{j,t}^{1}\\ \underset{j'\in J}{\sum }\frac{c_{j,j'}\left(\chi^{A}A_{j',t}+\chi^{P}P_{j',t}+\mathrm{IS}M_{j',t}\right)}{N_{j'}}-\alpha S_{j,t}^{1},\;\forall j\in J,t\in T \end{array}$$

(3c) $$\begin{array}{l} S_{j,t+1}^{2}=S_{j,t}^{2}+\alpha S_{j,t}^{1}-\left(1-\upsilon_{\inf}^{2}\right)\rho_{j}\beta_{t}S_{j,t}^{2}\\ \underset{j'\in J}{\sum }\frac{c_{j,j'}\left(\chi^{A}A_{j',t}+\chi^{P}P_{j',t}+\mathrm{IS}M_{j',t}\right)}{N_{j'}}-y_{j,t},\;\forall j\in J,t\in T \end{array}$$

(3d) $$\begin{array}{l} S_{j,t+1}^{3}=S_{j,t}^{3}+y_{j,t}-\left(1-\upsilon_{\inf}^{3}\right)\rho_{j}\beta_{t}S_{j,t}^{3}\\ \underset{j'\in J}{\sum }\frac{c_{j,j'}\left(\chi^{A}A_{j',t}+\chi^{P}P_{j',t}+\mathrm{IS}M_{j',t}\right)}{N_{j'}}-\alpha S_{j,t}^{3},\;\forall j\in J,t\in T \end{array}$$

(3e) $$\begin{array}{l} S_{j,t+1}^{4}=S_{j,t}^{4}+\alpha S_{j,t}^{3}-\left(1-\upsilon_{\inf}^{4}\right)\rho_{j}\beta_{t}S_{j,t}^{4}\\ \underset{j'\in J}{\sum }\frac{c_{j,j'}\left(\chi^{A}A_{j',t}+\chi^{P}P_{j',t}+\mathrm{IS}M_{j',t}\right)}{N_{j'}},\;\forall j\in J,t\in T \end{array}$$

##### 3.3.2.3. Exposed individuals

Equation (4a) represents the number of unvaccinated exposed individuals in age group *j* at the end of period *t* + 1 to be equal to the number of unvaccinated exposed individuals in age group *j* in the previous period, plus the number of unvaccinated susceptible individuals in age group *j* who transition to the exposed state due to contact with pathogens infection at the time period *t*, minus the number of unvaccinated exposed individuals in age group *j* who become infectious at the time period *t*, where 1/ω is the mean latent period.

(4a) $$\begin{array}{l} E_{j,t+1}^{0}=E_{j,t}^{0}+\rho_{j}\beta_{t}S_{j,t}^{0}\\ \underset{j'\in J}{\sum }\frac{c_{j,j'}\left(\chi^{A}A_{j',t}+\chi^{P}P_{j',t}+\mathrm{IS}M_{j',t}\right)}{N_{j'}}-\omega E_{j,t}^{0},\;\forall j\in J,t\in T \end{array}$$

Similarly, the variation in the number of exposed individuals vaccinated with the first dose (protection has yet to be realized), exposed individuals vaccinated with the first dose (protected by the vaccine effect of the first dose), exposed individuals vaccinated with the second dose (no improve the protection efficiency yet), and exposed individuals vaccinated with the second dose (protected by the full vaccine effect of the two doses) were described in equations (4b)–(4e), respectively. In addition to this, the total number of exposed individuals (including unvaccinated and vaccinated) in age group *j* at the end of period *t* + 1 was defined by Equation (4f).

(4b) $$\begin{array}{l} E_{j,t+1}^{1}=E_{j,t}^{1}+\left(1-\upsilon_{\inf}^{1}\right)\rho_{j}\beta_{t}S_{j,t}^{1}\\ \underset{j'\in J}{\sum }\frac{c_{j,j'}\left(\chi^{A}A_{j',t}+\chi^{P}P_{j',t}+\mathrm{IS}M_{j',t}\right)}{N_{j'}}-\alpha E_{j,t}^{1}-\omega E_{j,t}^{1},\;\forall j\in J,t\in T\text{.} \end{array}$$

(4c) $$\begin{array}{l} E_{j,t+1}^{2}=E_{j,t}^{2}+\left(1-\upsilon_{\inf}^{2}\right)\rho_{j}\beta_{t}S_{j,t}^{2}\\ \underset{j'\in J}{\sum }\frac{c_{j,j'}\left(\chi^{A}A_{j',t}+\chi^{P}P_{j',t}+\mathrm{IS}M_{j',t}\right)}{N_{j'}}+\alpha E_{j,t}^{1}-\omega E_{j,t}^{2},\;\forall j\in J,t\in T\text{.} \end{array}$$

(4d) $$\begin{array}{l} E_{j,t+1}^{3}=E_{j,t}^{3}+\left(1-\upsilon_{\inf}^{3}\right)\rho_{j}\beta_{t}S_{j,t}^{3}\\ \underset{j'\in J}{\sum }\frac{c_{j,j'}\left(\chi^{A}A_{j',t}+\chi^{P}P_{j',t}+\mathrm{IS}M_{j',t}\right)}{N_{j'}}-\alpha E_{j,t}^{3}-\omega E_{j,t}^{3},\;\forall j\in J,t\in T\text{.} \end{array}$$

(4e) $$\begin{array}{l} E_{j,t+1}^{4}=E_{j,t}^{4}+\left(1-\upsilon_{\inf}^{4}\right)\rho_{j}\beta_{t}S_{j,t}^{4}\\ \underset{j'\in J}{\sum }\frac{c_{j,j'}\left(\chi^{A}A_{j',t}+\chi^{P}P_{j',t}+\mathrm{IS}M_{j',t}\right)}{N_{j'}}+\alpha E_{j,t}^{3}-\omega E_{j,t}^{4},\;\forall j\in J,t\in T\text{.} \end{array}$$

(4f) $$E_{j,t+1}=E_{j,t+1}^{0}+\underset{k\in K}{\sum }E_{j,t+1}^{k},\;\forall j\in J,t\in T\text{.}$$

##### 3.3.2.4. Asymptomatic infectious individuals

Equation (5) describes the number of asymptomatic infectious individuals in age group *j* at the end of period *t* + 1 to be equal to the number of asymptomatic infectious individuals in age group *j* in the previous period, plus the total number of newly asymptomatic infectious individuals (including unvaccinated and vaccinated) in age group *j* transferred from exposed infectious individuals at the time period *t*, minus the number of the asymptomatic infectious individuals who recovered at the time period *t*, where $\sigma_{j}$ is the proportion of exposed individuals of age group *j* who become pre-symptomatic. The parameter $1/\eta^{AR}$ denotes the mean recovery time of asymptomatic infectious individuals. In addition, we use $\upsilon_{\mathrm{sympt}}^{k}$ to represent the vaccine effectiveness against symptomatic disease in the vaccination status *k*.


(5) $$\begin{array}{l} A_{j,t+1}=A_{j,t}+\left(1-\sigma_{j}\right)\omega E_{j,k}^{0}+\\ \underset{k\in K}{\sum }\left(1-\sigma_{j}\left(1-\upsilon_{\mathrm{sympt}}^{k}\right)\right)\omega E_{j,k}^{k}-\eta^{AR}A_{j,t},\;\forall j\in J,t\in T\text{.} \end{array}$$

##### 3.3.2.5. Pre-symptomatic infectious individuals

Equation (6a) provides the number of unvaccinated pre-symptomatic infectious individuals in age group *j* at the end of period *t* + 1 to be equal to the number of unvaccinated pre-symptomatic infectious individuals in age group *j* in the previous period, plus the number of newly unvaccinated pre-symptomatic infectious individuals in age group *j* transferred from exposed infectious individuals at the time period *t*, minus the number of unvaccinated pre-symptomatic infectious individuals in age group *j* who transition to the mildly-symptomatic infectious state at the time period *t*, where 1/φ is the mean duration of the pre-symptomatic infectious period.


(6a) $$P_{j,t+1}^{0}=P_{j,t}^{0}+\sigma_{j}\omega E_{j,t}^{0}-\varphi P_{j,t}^{0},\;\forall j\in J,t\in T\text{.}$$

Along these same lines, the variation in the number of pre-symptomatic infectious individuals vaccinated with the first dose (protection has yet to be realized), pre-symptomatic infectious individuals vaccinated with the first dose (protected by the vaccine effect of the first dose), pre-symptomatic infectious individuals vaccinated with the second dose (no improve the protection efficiency yet), and pre-symptomatic infectious individuals vaccinated with the second dose (protected by the full vaccine effect of the two doses) were defined in equations (6b)–(6e), respectively. We used $\alpha \omega /\left(\omega -\alpha \right)$ to model the transition rate of the change in vaccination status of the pre-symptomatic infectious individuals vaccinated with the first dose (protection has yet to be realized) or pre-symptomatic infectious individuals vaccinated with the second dose (no improve the protection efficiency yet). Equation (6f) determines the total number of pre-symptomatic infectious individuals (including unvaccinated and vaccinated) in age group *j* at the end of period *t* + 1.


(6b) $$\begin{array}{l} P_{j,t+1}^{1}=P_{j,t}^{1}+\sigma_{j}\left(1-\upsilon_{\mathrm{sympt}}^{1}\right)\omega E_{j,t}^{1}-\frac{\alpha \omega }{\omega -\alpha }P_{j,t}^{1}-\varphi P_{j,t}^{1},\;\\ \;\forall j\in J,t\in T\text{.} \end{array}$$

(6c) $$\begin{array}{l} P_{j,t+1}^{2}=P_{j,t}^{2}+\sigma_{j}\left(1-\upsilon_{\mathrm{sympt}}^{2}\right)\omega E_{j,t}^{2}+\frac{\alpha \omega }{\omega -\alpha }P_{j,t}^{1}-\varphi P_{j,t}^{2},\;\\ \;\forall j\in J,t\in T\text{.} \end{array}$$

(6d) $$\begin{array}{l} P_{j,t+1}^{3}=P_{j,t}^{3}+\sigma_{j}\left(1-\upsilon_{\mathrm{sympt}}^{3}\right)\omega E_{j,t}^{3}-\frac{\alpha \omega }{\omega -\alpha }P_{j,t}^{3}-\varphi P_{j,t}^{3},\;\\ \forall j\in J,t\in T\text{.} \end{array}$$

(6e) $$\begin{array}{l} P_{j,t+1}^{4}=P_{j,t}^{4}+\sigma_{j}\left(1-\upsilon_{\mathrm{sympt}}^{4}\right)\omega E_{j,t}^{4}+\frac{\alpha \omega }{\omega -\alpha }P_{j,t}^{3}-\varphi P_{j,t}^{4},\;\\ \forall j\in J,t\in T\text{.} \end{array}$$

(6f) $$P_{j,t+1}=P_{j,t+1}^{0}+\underset{k\in K}{\sum }P_{j,t+1}^{k},\;\forall j\in J,t\in T\text{.}$$

##### 3.3.2.6. Mildly-symptomatic infectious individuals

Equation (7a) represents the number of unvaccinated mildly-symptomatic infectious individuals in age group *j* at the end of period *t* + 1, equal to the number of unvaccinated mildly-symptomatic infectious individuals in age group *j* in the previous period, plus the number of newly mildly-symptomatic infectious individuals in age group *j* transferred from unvaccinated pre-symptomatic infectious individuals at the time period *t*, minus the number of unvaccinated mildly-symptomatic infectious individuals in age group *j* who transition to the severely-symptomatic infectious state at the time period *t*, minus the number of the unvaccinated mildly-symptomatic infectious individuals who recovered at the time period *t*, where $\varepsilon_{j}$ is the proportion of mildly-symptomatic infectious individuals of age group *j* who develop severe disease requiring hospitalization. The parameter 1/δ denotes the mean duration of the mildly-symptomatic infectious period, and the parameter $1/\eta^{\mathrm{ISMR}}$ represents the mean recovery time of mildly-symptomatic infectious individuals.


(7a) $$\begin{array}{l} \mathrm{IS}M_{j,t+1}^{0}=\mathrm{IS}M_{j,t}^{0}+\varphi P_{j,t}^{0}-\varepsilon_{j}\delta ISM_{j,t}^{0}-\left(1-\varepsilon_{j}\right)\eta^{\mathrm{ISMR}}\mathrm{IS}M_{j,t}^{0},\;\\ \forall j\in J,t\in T\text{.} \end{array}$$

Similarly, the variation in the number of mildly-symptomatic infectious individuals vaccinated with the first dose (protection has yet to be realized), mildly-symptomatic infectious individuals vaccinated with the first dose (protected by the vaccine effect of the first dose), mildly-symptomatic infectious individuals vaccinated with the second dose (no improve the protection efficiency yet), and mildly-symptomatic infectious individuals vaccinated with the second dose (protected by the full vaccine effect of the two doses) were described separately in equations (7b)–(7e). We used $\frac{\alpha \omega \varphi }{\omega \varphi -\alpha \varphi -\alpha \omega }$ to model the transition rate of the change in vaccination status of the mildly-symptomatic infectious individuals vaccinated with the first dose (protection has yet to be realized) or mildly-symptomatic infectious individuals vaccinated with the second dose (no improve the protection efficiency yet). The parameter $\upsilon_{sd}^{k}$ represents the vaccine effectiveness against severe disease in the vaccination status *k*. Equation (7f) determines the total number of mildly-symptomatic infectious individuals (including unvaccinated and vaccinated) in age group *j* at the end of period *t* + 1.

(7b) $$\begin{array}{c} \mathrm{IS}M_{j,t+1}^{1}=\mathrm{IS}M_{j,t}^{1}+\varphi P_{j,t}^{1}-\frac{\alpha \omega \varphi }{\omega \varphi -\alpha \varphi -\alpha \omega }\mathrm{IS}M_{j,t}^{1}\\ -\varepsilon_{j}\left(1-\upsilon_{sd}^{1}\right)\delta ISM_{j,t}^{1}-\left(1-\varepsilon_{j}\left(1-\upsilon_{sd}^{1}\right)\right)\eta^{\mathrm{ISMR}}\mathrm{IS}M_{j,t}^{1},\\ \;\forall j\in J,t\in T\text{.} \end{array}$$

(7c) $$\begin{array}{c} \mathrm{IS}M_{j,t+1}^{2}=\mathrm{IS}M_{j,t}^{2}+\varphi P_{j,t}^{2}+\frac{\alpha \omega \varphi }{\omega \varphi -\alpha \varphi -\alpha \omega }\mathrm{IS}M_{j,t}^{1}\\ -\varepsilon_{j}\left(1-\upsilon_{sd}^{2}\right)\delta ISM_{j,t}^{2}-\left(1-\varepsilon_{j}\left(1-\upsilon_{sd}^{2}\right)\right)\eta^{\mathrm{ISMR}}\mathrm{IS}M_{j,t}^{2},\;\\ \forall j\in J,t\in T\text{.} \end{array}$$

(7d) $$\begin{array}{c} \mathrm{IS}M_{j,t+1}^{3}=\mathrm{IS}M_{j,t}^{3}+\varphi P_{j,t}^{3}-\frac{\alpha \omega \varphi }{\omega \varphi -\alpha \varphi -\alpha \omega }\mathrm{IS}M_{j,t}^{3}\\ -\varepsilon_{j}\left(1-\upsilon_{sd}^{3}\right)\delta ISM_{j,t}^{3}-\left(1-\varepsilon_{j}\left(1-\upsilon_{sd}^{3}\right)\right)\eta^{\mathrm{ISMR}}\mathrm{IS}M_{j,t}^{3},\;\\ \forall j\in J,t\in T\text{.} \end{array}$$

(7e) $$\begin{array}{c} \mathrm{IS}M_{j,t+1}^{4}=\mathrm{IS}M_{j,t}^{4}+\varphi P_{j,t}^{4}+\frac{\alpha \omega \varphi }{\omega \varphi -\alpha \varphi -\alpha \omega }\mathrm{IS}M_{j,t}^{3}\\ -\varepsilon_{j}\left(1-\upsilon_{sd}^{4}\right)\delta ISM_{j,t}^{4}-\left(1-\varepsilon_{j}\left(1-\upsilon_{sd}^{4}\right)\right)\eta^{\mathrm{ISMR}}\mathrm{IS}M_{j,t}^{4},\;\\ \;\forall j\in J,t\in T\text{.} \end{array}$$

(7f) $$\mathrm{IS}M_{j,t+1}=\mathrm{IS}M_{j,t+1}^{0}+\underset{k\in K}{\sum }\mathrm{IS}M_{j,t+1}^{k},\;\forall j\in J,t\in T\text{.}$$

##### 3.3.2.7. Severely-symptomatic infectious individuals

Equation (8) describes the number of severely-symptomatic infectious individuals in age group *j* at the end of period *t* + 1, which is equal to the number of severely-symptomatic infectious individuals in age group *j* in the previous period plus the total number of newly severely-symptomatic infectious individuals (including unvaccinated and vaccinated) in age group *j* transferred from mildly-symptomatic infectious individuals at the time period *t*, minus the number of the severely-symptomatic infectious individuals in age group *j* who are admitted to hospital at the time period *t*, where 1/λ denotes the mean duration of severe infection prior to hospitalization.


(8) $$\begin{array}{c} \mathrm{IS}S_{j,t+1}=\mathrm{IS}S_{j,t}+\varepsilon_{j}\delta ISM_{j,t}^{0}+\underset{k\in K}{\sum }\varepsilon_{j}\left(1-\upsilon_{\int sd}^{k}\right)\delta ISM_{j,t}^{k}-\lambda ISS_{j,t},\\ \;\forall j\in J,t\in T\text{.} \end{array}$$

##### 3.3.2.8. Infected-hospitalized cases in the ICU

According to Equation (9), the number of infected-hospitalized cases in the ICU for age group *j* at the end of period *t* + 1 is equal to the number of infected-hospitalized cases in the ICU for age group *j* in the previous period plus the number of newly infected-hospitalized cases in the ICU transferred from severely-symptomatic infectious individuals for age group *j* at the time period *t*, minus the number of infected-hospitalized cases in the ICU for age group *j* who recovered or died while on therapy at the time period *t*, where $\psi_{j}$ is the proportion of hospitalized cases of age group *j* who required ICU and $\gamma_{j}$ is the proportion of ICU cases of age group *j* who died. The parameter $1/\eta_{t}^{\mathrm{ICUR}}$ represents the mean recovery time of ICU cases at time period *t*, and the parameter $1/\zeta_{t}^{\mathrm{ICUD}}$ denotes the mean time of ICU cases who died at time period *t.*

(9) $$\begin{array}{c} ICU_{j,t+1}=ICU_{j,t}+\psi_{j}\lambda ISS_{j,t}\\ -\left(1-\gamma_{j}\right)\eta_{t}^{\mathrm{ICUR}}ICU_{j,t}-\gamma_{j}\zeta_{t}^{\mathrm{ICUD}}ICU_{j,t},\;\\ \;\forall j\in J,t\in T\text{.} \end{array}$$

##### 3.3.2.9. Infected-hospitalized cases in the general ward

Equation (10) implies that the number of infected-hospitalized cases in the general ward for age group *j* at the end of period *t* + 1 equals the number of infected-hospitalized cases in the general ward for age group *j* in the previous period plus the number of newly infected-hospitalized cases in the general ward transferred from severely-symptomatic infectious individuals for age group *j* at the time period *t*, minus the number of infected-hospitalized cases in the general ward for age group *j* who recovered or died while on therapy at the time period *t*, where $\theta_{j}$ is the proportion of non-ICU cases of age group *j* who died. The parameter $1/\eta_{t}^{HR}$ denotes the mean recovery time of non-ICU cases at time period *t*, and the parameter $1/\zeta_{t}^{HD}$ represents the mean time of non-ICU cases who died at time period *t.*

(10) $$\begin{array}{l} H_{j,t+1}=H_{j,t}+\left(1-\psi_{j}\right)\lambda ISS_{j,t}\\ -\left(1-\theta_{j}\right)\eta_{t}^{HR}H_{j,t}-\theta_{j}\zeta_{t}^{HD}H_{j,t},\;\forall j\in J,t\in T\text{.} \end{array}$$

##### 3.3.2.10. Recovered individuals

Equation (11) provides the total number of recovered individuals for age group *j* at the end of period *t* + 1, which is equal to the number of recovered individuals for age group *j* in the previous period, plus the newly recovered individuals for age group *j* from asymptomatic infectious individuals, mildly-symptomatic infectious individuals, infected-hospitalized cases in the ICU, and infected-hospitalized cases in the general ward at the time period *t*, minus the number of recovered individuals in age group *j* who transition to the fully susceptible state owing to the waning of infection-derived temporary immunity at the time period *t*.

(11) $$\begin{array}{l} R_{j,t+1}=R_{j,t}+\eta^{AR}A_{j,t}+\left(1-\varepsilon_{j}\right)\eta^{\mathrm{ISMR}}\mathrm{IS}M_{j,t}^{0}\\ +\underset{k\in K}{\sum }\left(1-\varepsilon_{j}\left(1-\upsilon_{sd}^{k}\right)\right)\eta^{\mathrm{ISMR}}\mathrm{IS}M_{j,t}^{k}+\left(1-\gamma_{j}\right)\eta_{t}^{\mathrm{ICUR}}ICU_{j,t}\\ +\left(1-\theta_{j}\right)\eta_{t}^{HR}H_{j,t}-\tau R_{j,t},\;\forall j\in J,t\in T \end{array}$$

##### 3.3.2.11. Deceased individuals

Equation (12) shows that the total number of deceased individuals for age group *j* at the end of period *t* + 1 is equal to the sum of (i) the number of deceased individuals for age group *j* in the previous period; (ii) the number of infected-hospitalized cases in the ICU for age group *j* who died at the time period *t*; and (iii) the number of infected-hospitalized cases in the general ward for age group *j* who died at the time period *t*.

(12) $$D_{j,t+1}=D_{j,t}+\gamma_{j}\zeta_{t}^{\mathrm{ICUD}}ICU_{j,t}+\theta_{j}\zeta_{t}^{HD}H_{j,t},\;\forall j\in J,t\in T\text{.}$$

#### 3.3.3. Vaccine resources allocation constraints

In this subsection, we presented constraints (13)–(17) related to the allocation and logistics operations management of limited vaccine available, which are simultaneously optimized under a dynamic environment of the epidemic described above by Equations (2)–(12) in our model.

(13) $$\underset{j\in J}{\sum }\left(x_{j,t}+y_{j,t}\right)\leq C_{t},\;\forall t\in T\text{,}$$

(14) $$C_{t+1}=B_{t+1}+\left(C_{t}-\underset{j\in J}{\sum }\left(x_{j,t}+y_{j,t}\right)\right),\;\forall t\in T\text{,}$$

(15) $$x_{j,t}\leq S_{j,t}^{0},\;\forall j\in J,t\in T\text{,}$$

(16) $$\frac{\alpha \varsigma_{\mathrm{interval}}^{U}}{\alpha -\varsigma_{\mathrm{interval}}^{U}}S_{j,t}^{2}\leq y_{j,t}\leq \frac{\alpha \varsigma_{\mathrm{interval}}^{L}}{\alpha -\varsigma_{\mathrm{interval}}^{L}}S_{j,t}^{2},\;\forall j\in J,t\in T\text{,}$$

(17) $$x_{j,t}=0,y_{j,t}=0,\;\forall j\in \left\{0-4,5-9,10-14\right\},t\in T\text{.}$$

Constraint (13) ensures that the total number of the first- and second-dose administered cannot exceed the available vaccine capacities at the time period *t*. Constraint (14) determines the total available vaccine capacities at the time period *t* + 1 equals the number of vaccine doses supplied at time period *t* + 1 plus the number of unused vaccines in the previous period. Constraint (15) guarantees that the number of vaccines allocated to individuals who administer the first dose is not more than the number of unvaccinated susceptible individuals in age group *j* at the time period *t*. Constraint (16) limits the second dose should be administered in the specific time windows after the first dose, where $1/\varsigma_{\mathrm{interval}}^{U}$ and $1/\varsigma_{\mathrm{interval}}^{L}$ represent the maximum and minimum time interval between the administration of the first dose and second dose, respectively. Constraint (17) imposes an age restriction for universal COVID-19 vaccination because the majority of COVID-19 vaccines are approved for use above 15 years old at the beginning of the vaccine rollout (71).

#### 3.3.4. Non-negativity constraints

Constraint (18) shows that all variables should be greater or equal to zero.

(18) Allvariablesarecontinuousandnon−negative,∀j∈J,t∈T.

Finally, we observe that Equations (3a)–(4e) are nonlinear and non-convex due to the presence of bi-linear terms. Therefore, the proposed model in Equations (1)–(18) is a nonlinear programming formulation for the multi-period two-dose vaccine allocation problem. In the next section, we applied the above proposed NLP model in a case study involving vaccination of the 2021 COVID-19 in the Midlands of England to illustrate the usefulness of our model.

## 4. Case study

England is among the countries most severely affected by the COVID-19 pandemic since the first cases of COVID-19 were reported on January 31, 2020 (14). Although some interventions, such as maintaining safe social distancing, contact tracing, and testing of suspect cases were implemented to suppress the early spread of the virus, it was insufficient to control the epidemic in England. In response to this public health emergency, several COVID-19 vaccines have been fast developed and approved through a global collaborative effort between many scientists and were deployed first in England on December 8, 2020 (17). According to the National Health Service (NHS) division, England is composed of seven NHS regions. We select the Midlands of England that the most populous for our case study. Here, we focused on the application of the NLP model proposed in Section 3 to determine the optimal allocation of a limited vaccine resource and to minimize the total number of deaths in the Midlands of England.

We provide the case study data used in the model, including epidemiological parameters, population, initial conditions data, and vaccine efficacy data. The corresponding epidemiological parameters are summarized in Supplementary Tables 1, 2. These parameters are primarily from a series of references and public sources, as cited in the table. Supplementary Table 3 presents the contact matrix between different age groups, which was derived from the POLYMOD survey (72) about contacts relevant for the transmission of diseases for the United Kingdom using the ‘socialmixr’ R package (73), scaling the Midlands of England population demography data to obtain the required daily age-group-specific contact matrix. Furthermore, the time-varying transmission rate 𝛽*_t_* that captures the transmission efficiency as a function of time due to changes in COVID-19 policy. In our case study, the time-varying transmission rate was obtained from Sonabend et al. (17), which mainly focused on the key epidemiological drivers of COVID-19 in each NHS England region.

Supplementary Table 4 presents information about the population size and age distribution from the Office for National Statistics (ONS) mid-2020 population estimates for the Midlands of England (74). In our case study, we consider a 25 weeks vaccination campaign that began on December 8, 2020, that is, the start time of the vaccination campaign in the Midlands of England. An important data set is the initial condition of the epidemic at the start of the planning horizon. We set the initially infected individuals to 1.21% of the total population in the region, which can be obtained from the ONS coronavirus infection survey regarding the number of people testing positive for COVID-19 within the community population during December 6, 2020, to December 12, 2020, in the Midlands of England (75). We estimated the initial number of infected individuals from each age group by multiplying the total infected individuals by the proportion initially of different age groups from the official United Kingdom government website for data on coronavirus (76). Of these, the number of severely-symptomatic infectious individuals is directly proportional to the number of new infected-hospitalized cases at that time. Therefore, the new infected-hospitalized cases data from GOV.UK can be used to get an approximation of the initial number of severely-symptomatic infectious individuals. In addition, we assume the initial number of remaining infected individuals is assigned homogeneously among the different types of infections, including exposed, asymptomatic infectious, pre-symptomatic infectious, and mildly-symptomatic infectious individuals. Similarly, we set the initially recovered individuals to 9.03% of the total population in the region, which is available from the ONS an estimated of people would have tested positive for antibodies against COVID-19 on a blood test in the Midlands of England in early December 2020 (77). We estimated the initial number of recovered individuals from each age group by multiplying the total recovered individuals by the proportion initially of different age groups (60). Besides this, we initialized the number of deceased individuals, infected-hospitalized cases in the general ward, and infected-hospitalized cases in the ICU using data reported from the GOV.UK on December 8, 2020. On this basis, we estimated the initial number of individuals of these three populations from each age group based on the corresponding proportion initially of different age groups (see Supplementary Table 4). Subsequently, the initial number of susceptible individuals was obtained by subtracting the initial number of infected, infected-hospitalized cases, recovered, and deceased individuals from the population size in the region. Finally, Supplementary Table 5 summarizes the vaccine efficacy against infection, symptomatic disease, and severe disease based mostly on previously published literature.

## 5. Results and discussions

In this section, we first validated the proposed model against real-world data from the COVID-19 outbreak in the Midlands of England. Then, we conduct comparative studies to evaluate the performance of our optimal strategy with different strategies, which may be derived from the existing literature. Finally, we elaborate on the numerical results and discussions obtained by solving our model with respect to different settings of vaccine supply levels and the start time of vaccination. Our mathematical formulations were coded in the Julia using the JuMP (78) modeling language and solved using the Interior Point Optimizer (IPOPT) (79) with the MA97 parallel linear solver (80). All computational experiments were run on a desktop computer equipped with LINUX operating system with 8 cores, 1.8 gigahertz CPU, and 32-gigabyte memory.

### 5.1. Model validation

The proposed mathematical model is validated against the officially reported pandemic data in the Midlands of England to accurately predict the progress of the COVID-19 epidemic from December 8, 2020, to May 30, 2021, within a given parameters settings. Specifically, the proposed model is solved with fixed decision variable values based on real vaccination data from GOV.UK database. Afterward, we compared the predicted outbreak data with respect to the cumulative number of deaths, hospital admissions, and hospital bed occupancy by our model to the actual outbreak data given in the Public Health England (76).

**Figure 2:**
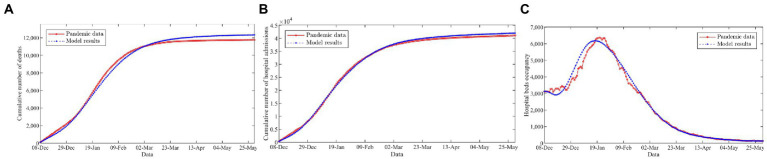
Comparison of the officially reported pandemic data and the model results. **(A)** Cumulative number of deaths. **(B)** Cumulative number of hospital admissions. **(C)** Hospital beds occupancy.

Figure 2 gives an intuitive comparison between the officially reported pandemic data (red circles, solid line) and the prediction results of the model (blue asterisks, dashed line). Figure 2 indicates that the proposed model provides an excellent fit for the cumulative number of deaths, hospital admissions, and hospital bed occupancy. In addition, we further assess the performance of the model using three common metrics, including the mean absolute percentage error (MAPE), the normalized root mean squared error (*n*RMSE), and the explained variance between the officially reported pandemic data and the prediction results of the model, as illustrated in Table 1. The results show that the MAPE and *n*RMSR values are quite low and explained variance values close to 1. This implies that the model is reliable and could be used to characterize the transmission dynamics of the disease outbreak.

**Table 1:** Statistical analysis to compare the officially reported pandemic data and the model results.

Data	Metric		
	Mean absolute percentage error (%)	Normalized root mean squared error	Explained variance (%)
Cumulative number of deaths	5.23	0.0425	99.13
Cumulative number of hospital admissions	3.03	0.0203	99.83
Hospital beds occupancy	5.07	0.0980	98.79

### 5.2. Vaccine resources allocation based on the optimal solution

In this subsection, we show that the optimal allocation strategy by solving the multi-period two-dose vaccine allocation model with an application to the COVID-19 vaccination campaign in the Midlands of England using the data described in the previous section. Supplementary Figure 1 presents the number of the first and second doses of vaccine were allocated per day. We observe that the daily number of vaccine doses supplied began at approximately 2,000, rapidly increased to over 50,000 by January 8, 2021, and reached a peak value of more than 150,000 on March 20, 2021. Furthermore, we found that the vast majority of vaccine resources were used to administer the first dose to improve vaccine coverage during the first 2 months of vaccination campaigns. As a growing number of vaccine resources become available, more vaccines will have used to cover more populations with the full two doses of vaccine in order to confer adequate protection. As shown in Supplementary Figure 2, by February 8, 2021, roughly 17.6% of the total population will have been vaccinated with at least one dose and only 2.4% have been vaccinated with two doses, followed by a gradual increase to 58.2% (first dose) and 38.3% (second dose) by May 30, 2021, according to the optimal allocation strategy.

**Figure 3:**
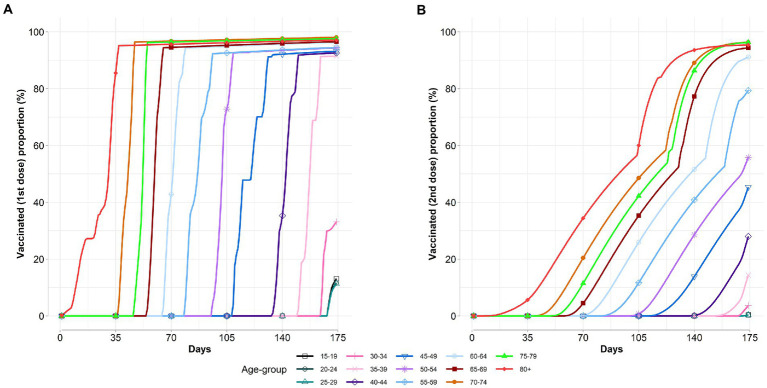
Age-specific vaccinated proportions vary over time. **(A)** Vaccinated first dose proportion. **(B)** Vaccinated second dose proportion.

Figure 3 shows the vaccine coverage varies over time per age group. According to the results, we observe that the optimal allocation strategy is dynamic and is specific for a targeting vaccination group in each period. Broadly, we find that the vaccination strategies prioritized the elderly populations, and then the vaccine rollout was extended to younger age groups as more vaccines became available over time. Moreover, we also find that a new age group is added to the campaign before 100% vaccination coverage of the previous age group is performed. This is due to the diminishing marginal effects of additional vaccination before 100% vaccination coverage of the previous age group caused the vaccine rollout switch to the new age group.

### 5.3. Comparative studies

We compare our optimal vaccine allocation strategy with 12 alternative strategies. These alternative strategies are derived from a combination of different two-dose vaccine rollout policies and different vaccine priority rules.

1. The two-dose vaccine rollout policies

- Hold-back policy: a COVID-19 vaccine rollout policy was originally implemented by the United States government (81). Specifically, one extra dose was stored in the storage room when a recipient received the first vaccine dose, and this dose will be used for this recipient once they come back to get their second dose. In other words, in each period, only half of the available vaccine capacities were used for the first dose of vaccination, and the other half was stored to address the future of the second vaccination.
- Release policy: another COVID-19 vaccine rollout policy in the United States, which Then–President-Elect Joe Biden announced that it would accelerate the available vaccine release to inoculate more individuals on January 8, 2021, thereby displacing the originally Hold-back policy (82). Concretely, the available vaccine capacities were administered either the first dose for new recipients or the second dose for returning recipients. In addition, the release policy requires doses first used by individuals who had already received the first dose and are eligible for a second dose; the remaining doses were used for the first dose of vaccination (83).
- Dose-stretching policy: the United Kingdom was the first to implement this COVID-19 vaccine rollout policy (17). This policy is similar to the release policy, except that it stretches the lead time for the second dose vaccination. In brief, the dose-stretching policy was no longer immediately provides the vaccine dose for the individuals who eligible for a second dose but stretches the lead time for the second dose vaccination as much as possible on the premise of guaranteeing the first dose of the vaccine without an immunity failure.

1. The vaccine priority rules

We also considered four vaccine priority rules in which the doses were allocated to each age group:

- Oldest first: prioritizes the allocation of vaccines to the oldest age groups first and then to younger age groups.
- Youngest first: prioritizes the allocation of vaccines to the youngest age groups first and then to older age groups.
- Pro-rata: vaccines are allocated proportionally to the size of the population within each age group.
- Uniform: vaccines are allocated uniformly in all age groups.

To facilitate comparisons, we included the no-vaccination scenario as a benchmark here. Supplementary Figure 3 shows the trajectories of the cumulative number of deaths with respect to various vaccine allocation strategies. As expected, any vaccine allocation strategy significantly reduces the number of deaths by 50–70% than the no-vaccination benchmark. From Supplementary Figures 3A,B, we observe that our optimal strategy performs better than the other 12 strategies in terms of saving more lives. The result of the dose-stretching policy (oldest first) is the closest to our optimal strategy, followed by the release policy (oldest first), whereas the hold-back policy (youngest first) is the worst. Furthermore, it is shown that with the same vaccine priority rule, the release policy (i.e., 71.3, 54.2, 63.5, and 64.1% deaths averted for the oldest first, youngest first, pro-rate, and uniform allocation strategy, respectively) and dose-stretching policy (i.e., 72.4, 52.9, 64.0, and 65.4%, respectively) outperforms the hold-back policy (i.e., 68.0, 49.0, 59.8, and 60.5%, respectively). This is probably because more people will receive their first dose under the release policy and dose-stretching policy, and thus the vaccine-induced immunity will work earlier, thereby slowing down the virus spread in the early stage of the vaccine rollout. On the other hand, we also observed that under the same two-dose vaccine rollout policy, the “oldest first” rule is the most effective in deaths averted, followed by the uniform and pro-rata allocation, and the worst is the “youngest first” rule. The result of the simulation suggests that the high-risk group should be given higher priority during vaccine promotion in order to avert more deaths.

### 5.4. Impact of the level of supply on vaccine allocation and disease progression

In this subsection, we further explored the impact of the level of supply on the allocation of vaccine resources and the course of the epidemic. Concretely, we investigate the optimal vaccine allocation results and disease progression for seven counterfactual scenarios based on the different supply levels: without vaccines, 15,000, 30,000, 45,000, 60,000, 75,000, and 90,000 doses/day. Supplementary Figure 4 summarizes the optimal vaccine allocation strategies for various supply levels. Similar to the results of the previous section, vaccine resources should be prioritized for older populations to mitigate the impacts of the pandemic in terms of the number of deaths.

**Figure 4:**
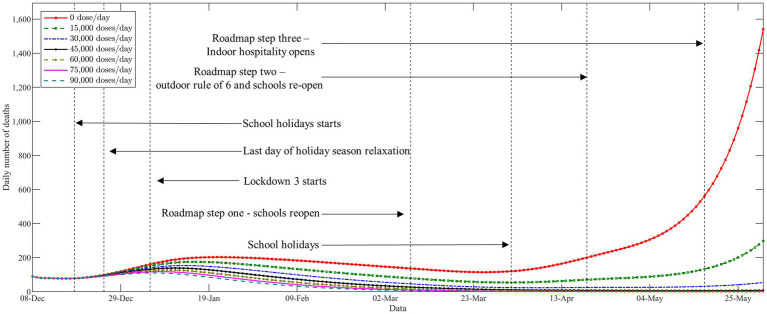
The trajectories of the daily number of deaths with respect to different levels of vaccine supply.

In Figure 4, we demonstrate the trajectories of the daily number of deaths with respect to different levels of vaccine supply. The vertical black dashed lines represent the time points to major changes in COVID-19 non-pharmaceutical interventions. As expected, the vaccination campaign plays an active role in terms of slowing the epidemic even at lower vaccine supply levels. It can be seen from Figure 4 that a new pandemic wave in the Midlands of England since the end of the second national lockdown on December 2, 2020. Shortly thereafter, the United Kingdom authorities announced to impose of a third national lockdown on January 5, 2021, to contain the rapid spread of the outbreak. We find that the epidemic came under good control during the third national lockdown, even in the absence of vaccine resources. The curves of the daily deaths with respect to different levels of vaccine supply started to visibly diverge since the third national lockdown was gradually eased during March 2021. It can be clearly seen that the epidemic resurgence and deaths have increased exponentially in the absence of vaccines. In addition, at low vaccine supply levels (below 15,000 doses per day), full relaxation would also lead to a new wave of deaths. Our results suggest that implementing a strict non-pharmaceutical intervention is necessary when there is an absence of vaccines. Moreover, the results also emphasize that the appropriate non-pharmaceutical intervention should be maintained throughout the entire vaccine rollout, especially in a low-supply scenario.

Finally, we compare the number of cumulative deaths during the studied horizon with respect to different levels of vaccine supply. As anticipated, more deaths averted can be observed when more vaccine doses are available. We also observe from Supplementary Figure 5 that the decline in deaths becomes less significant when the level of vaccine supply becomes high. This was somewhat expected due to the diminishing marginal benefit of vaccination when a high proportion of the population developed immunity. Furthermore, we further compare the performance of the different vaccine allocation strategies with respect to different levels of vaccine supply. As shown in Table 2, the experiments obtained similar results to those described in the previous section (see Section 5.3).

**Table 2:** Comparison of different vaccine allocation strategies with respect to different levels of vaccine supply.

	Vaccine allocation strategy	Level of vaccine supply					
		15,000 doses/day	30,000 doses/day	45,000 doses/day	60,000 doses/day	75,000 doses/day	90,000 doses/day
Cumulative number of deaths	Optimal	19,279	11,852	8,776	7,243	6,293	5,635
	Hold-back policy (oldest first)	22,275	14,740	11,316	9,493	8,362	7,556
	Hold-back policy (youngest first)	32,008	24,682	19,883	16,899	15,134	14,023
	Hold-back policy (pro-rate)	26,053	18,490	14,900	12,988	11,803	10,962
	Hold-back policy (uniform)	25,853	18,257	14,665	12,751	11,560	10,711
	Release policy (oldest first)	20,577	13,037	9,736	7,998	6,902	6,122
	Release policy (youngest first)	30,546	22,652	17,870	15,129	13,566	12,551
	Release policy (pro-rate)	24,290	16,673	13,279	11,488	10,349	9,516
	Release policy (uniform)	24,093	16,447	13,047	11,248	10,098	9,254
	Dose-stretching policy (oldest first)	19,298	11,854	8,794	7,396	6,462	5,757
	Dose-stretching policy (youngest first)	29,504	21,176	16,669	15,779	15,125	13,540
	Dose-stretching policy (pro-rate)	24,593	16,768	13,171	11,255	10,044	9,169
	Dose-stretching policy (uniform)	23,558	15,853	12,464	10,670	9,517	8,668
Averted proportion of deaths (%)	Optimal	54.72	72.16	79.39	82.99	85.22	86.77
	Hold-back policy (oldest first)	47.69	65.38	73.42	77.70	80.36	82.25
	Hold-back policy (youngest first)	24.83	42.03	53.30	60.31	64.46	67.07
	Hold-back policy (pro-rate)	38.81	56.57	65.01	69.50	72.28	74.25
	Hold-back policy (uniform)	39.28	57.12	65.56	70.05	72.85	74.84
	Release policy (oldest first)	51.67	69.38	77.13	81.22	83.79	85.62
	Release policy (youngest first)	28.26	46.80	58.03	64.47	68.14	70.52
	Release policy (pro-rate)	42.95	60.84	68.81	73.02	75.69	77.65
	Release policy (uniform)	43.42	61.37	69.36	73.58	76.28	78.27
	Dose-stretching policy (oldest first)	54.68	72.16	79.35	82.63	84.82	86.48
	Dose-stretching policy (youngest first)	30.71	50.27	60.85	62.94	64.48	68.20
	Dose-stretching policy (pro-rate)	42.24	60.62	69.07	73.57	76.41	78.47
	Dose-stretching policy (uniform)	44.67	62.77	70.73	74.94	77.65	79.64

### 5.5. Impact of the start time of vaccination on vaccine allocation and disease progression

In addition to the levels of vaccine supply, the start time of vaccination is another important factor that determines the performance of the vaccination campaign. In this subsection, we investigate the optimal vaccine allocation results and disease progression for different counterfactual scenarios based on the different start times of vaccination: delayed by day 0, day 15, day 30, day 45, and day 60 relative to December 8, 2020. Specifically, the model proposed is solved for several scenarios consisting of different levels of vaccine supply (from 15,000 to 9,000 doses/day as described in the above subsection) and the start time of vaccination. Supplementary Figures 6, 7 illustrates the age-specific vaccinated (first dose and second dose) proportions at the end of the first 5, 10, 15, 20, and 25 weeks with respect to different levels of vaccine supply and start time of vaccination, respectively. As can be seen in the figure, irrespective of scenarios, the elderly were consistently the priority candidates for vaccination.

Supplementary Figure 8 summarizes the cumulative number of deaths during the studied horizon with respect to different levels of vaccine supply and start time of vaccination. As seen in Supplementary Figure 8, longer delays in vaccination would produce more deaths under the same vaccine supply level. In addition, we also observed that delaying the start time of vaccination may drive worse outcomes when compared to significantly reducing the supply level of the vaccine but implementing vaccination campaigns as fast as possible. For example, providing 30,000 doses/day for vaccination on day 0 will result in 11,852 cases of deaths. However, compared to providing 75,000 doses/day and 90,000 doses/day for vaccination on day 60, that number will rise to 16,261 and 15,635, respectively. These results suggest that a vaccination campaign should be conducted as soon as possible in response to an epidemic outbreak once a safe and reliable vaccine has been successfully developed.

## 6. Conclusions and future study

In this paper, we present a novel multi-period two-dose vaccine allocation model for infectious disease control in the context of limited supply. Our model incorporates the transmission of infectious disease with the allocation and management of scarce vaccine resources with the objective to minimize the total number of deaths in a given population over the finite planning horizon. This model explicitly considers several key realistic features of the vaccine rollout process, such as the minimum and maximum time interval between the administration of the first and second dose should be complied with, the delayed onset of dose-specific immune responses, and multiple mechanisms of action of the vaccine. We have demonstrated the effectiveness of the proposed optimization model on a case study for the 2021 COVID-19 vaccination campaign in the Midlands of England and explored a wide range of plausible scenarios with respect to different levels of vaccine supply and start time of vaccination. We find that it is optimal to allocate vaccines to older age groups first is a robust strategy to avoid more deaths. Moreover, we also observed that releasing more vaccine doses for first-time users would provide an even larger vaccination benefit relative to holding back second doses. Our numerical results underscore the necessity of maintaining appropriate non-pharmaceutical intervention measures during vaccine rollout, particularly in low-resource settings. In addition, it is found that when the vaccine resources are limited but are currently available, starting vaccination as soon as possible provides significant benefits for mitigating the epidemic. The proposed approach is sufficiently generic and flexible and can be easily extended to other countries and regions to identify optimal vaccine allocation strategies for controlling epidemic spreading according to the available data.

Our research has several limitations, which should be considered in the future study. First, our model has only divided the population by age. However, other features, such as sex, occupation, health, geographic region, and race/ethnicity are also essential demographic classification factors in the human social structure. We believe that considering these demographic features will further improve the performance and applicability of the model. Second, we considered all vaccine-eligible individuals were willing to be vaccinated, this is a strong assumption. In the Supplementary Material, we relax this assumption, and a sensitivity analysis was performed to evaluate the effect of vaccine hesitancy. Third, one possible future research direction is to extend the proposed model to take into account the uncertainty of infectious disease transmission, e.g., the rates of symptom development, admission, and mortality. Fourth, although we chose the minimization of deaths as the objective function in this model, other public health objectives of vaccination, such as minimization of years of life lost, hospitalization, or infections are just as important, however, and deserve further exploration. Fifth, the proposed model does not take variants of COVID-19 into consideration. Therefore, our model may need recalibrations to be able to cope with the pandemic caused by new variants or strains, by means of adjusting some epidemiological parameters, which accordingly differ with variants of COVID-19. Sixth, our model has only been validated in a case study regarding the 2021 COVID-19 vaccination campaign in the Midlands of England. In the Supplementary Material, we further explored the application of the proposed model in two distinct National Health Service (NHS) regions. However, the generalization and the external validity of the model to other regions still need further investigation. Furthermore, the proposed vaccine allocation model is able to further generalize from the primary (two doses) to a more realistic scenario that contains the booster (third dose). Lastly, our study only focuses on a single region, it would be interesting to extend our method to incorporate multiple geographical regions.

## Data availability statement

The original contributions presented in the study are included in the article/Supplementary material, further inquiries can be directed to the corresponding author.

## Author contributions

JZ: conceptualization, methodology, software, formal analysis, visualization, and writing—original draft. QW: investigation, and writing—review and editing. MH: conceptualization, investigation, and supervision. All authors contributed to the article and approved the submitted version.

## Funding

This work is supported by the NSFC Major International (Regional) Joint Research Project Grant No. 71620107003; the Liaoning Revitalizing Talent Program No. XLYC1902010; the Fundamental Research Funds for State Key Laboratory of Synthetical Automation for Process Industries Grant No. 2013ZCX11; the 111 Project 2.0 (No. B08015).

## Conflict of interest

The authors declare that the research was conducted in the absence of any commercial or financial relationships that could be construed as a potential conflict of interest.

## Publisher’s note

All claims expressed in this article are solely those of the authors and do not necessarily represent those of their affiliated organizations, or those of the publisher, the editors and the reviewers. Any product that may be evaluated in this article, or claim that may be made by its manufacturer, is not guaranteed or endorsed by the publisher.
