# Supplementary material for: Optimizing two-dose vaccine resource allocation to combat a pandemic in the context of limited supply: The case of COVID-19
Source: Front Public Health. 2023 Apr 24;11:1129183. doi: 10.3389/fpubh.2023.1129183 (PMC10166111; doi:10.3389/fpubh.2023.1129183)
Supplement: Supplementary file 1 [file Data_Sheet_1.docx]

Supplementary Material

Optimizing two-dose vaccine resource allocation to combat a pandemic in the context of limited supply: The case of COVID-19

Jin Zhu, Qing Wang, Min Huang^*^

*** Correspondence:** Min Huang: mhuang@mail.neu.edu.cn

**Appendix A. Additional figures cited in the main manuscript**


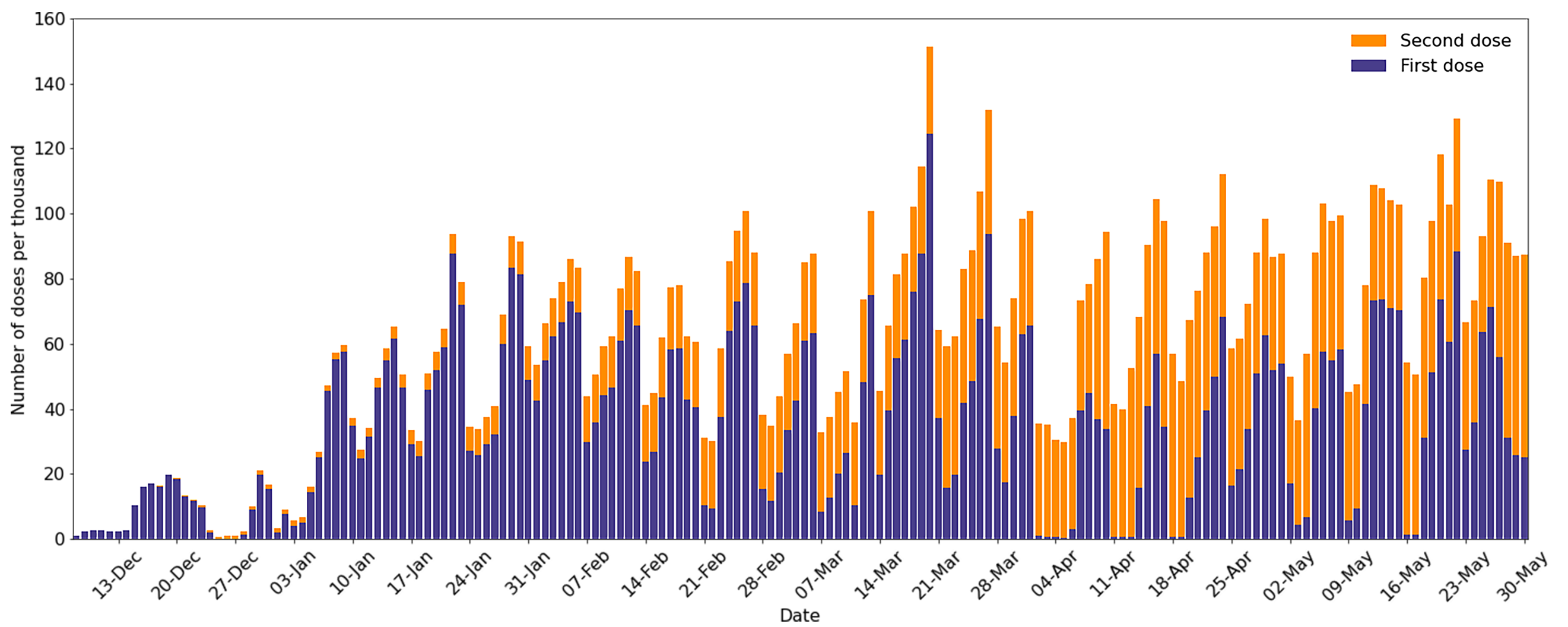


**Supplementary Figure 1.** Number of daily allocated vaccine doses.


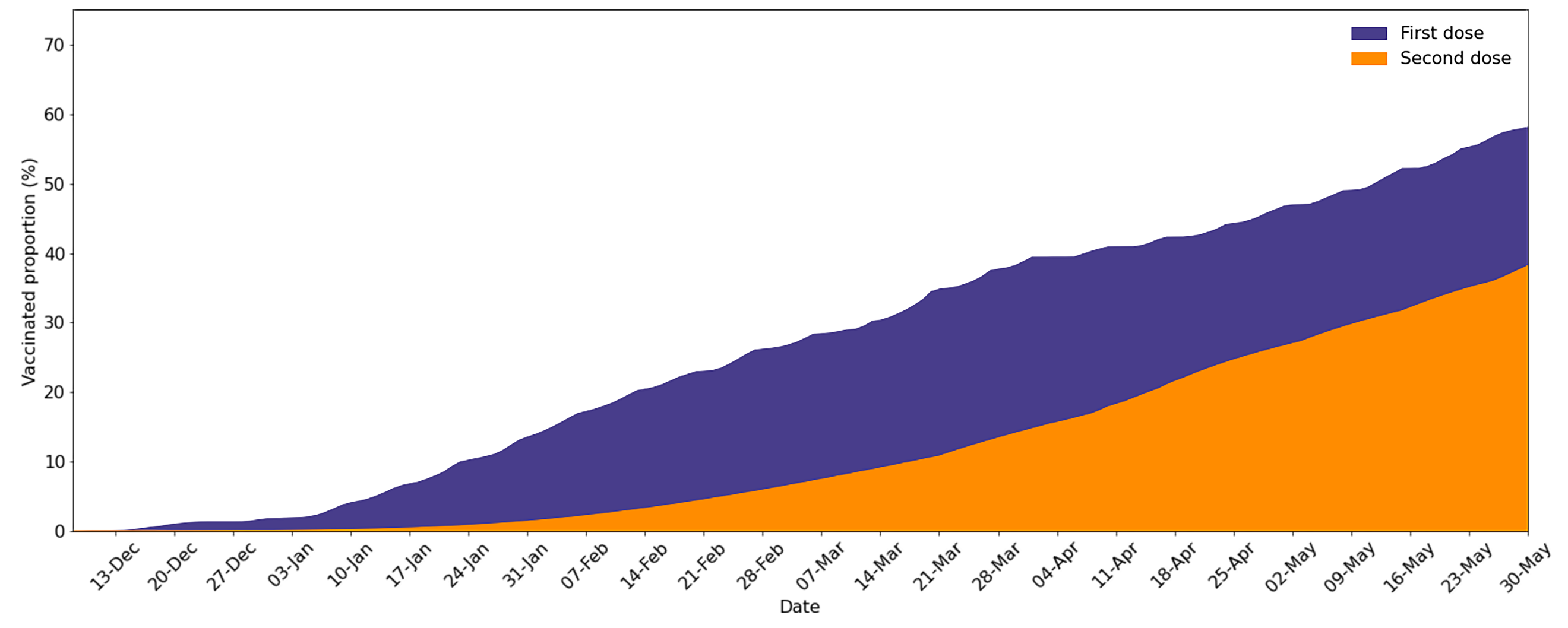


**Supplementary Figure 2.** Vaccinated proportion varies over time.


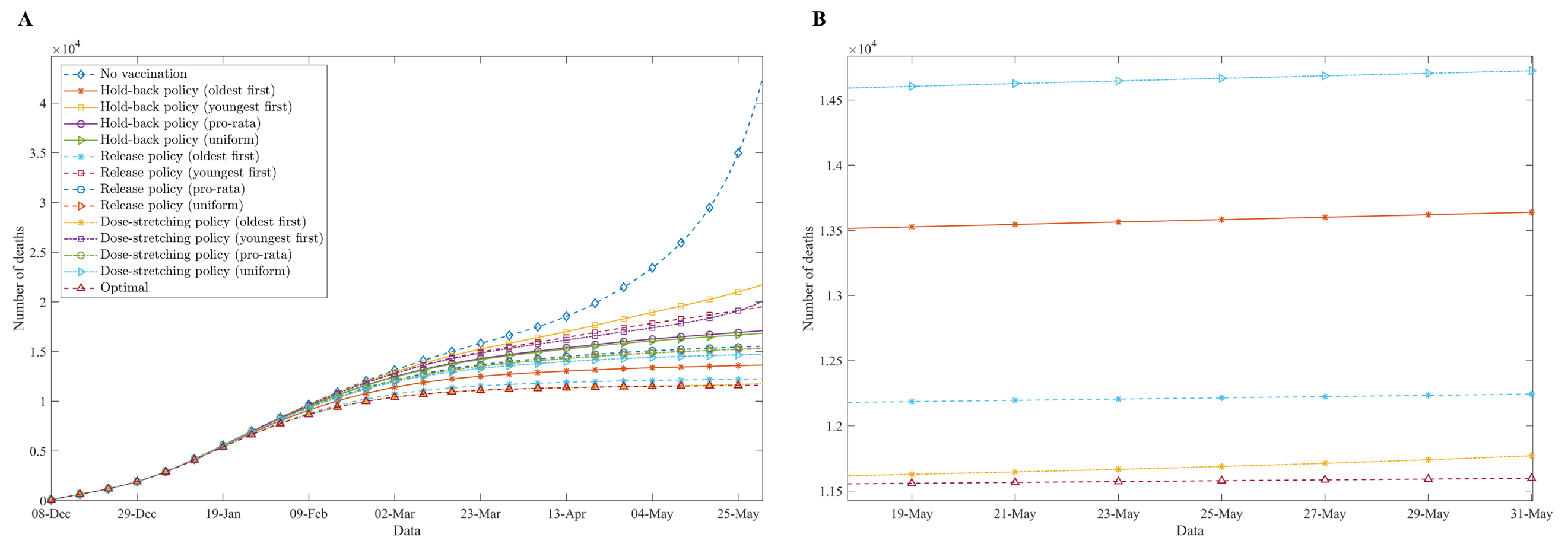


**Supplementary Figure 3.** The trajectories of the cumulative number of deaths with respect to various vaccine allocation strategies. **(A)** Cumulative number of deaths. **(B)** Zoom-in cumulative number of deaths, which shows the results from 19 May to 31 May 2021.


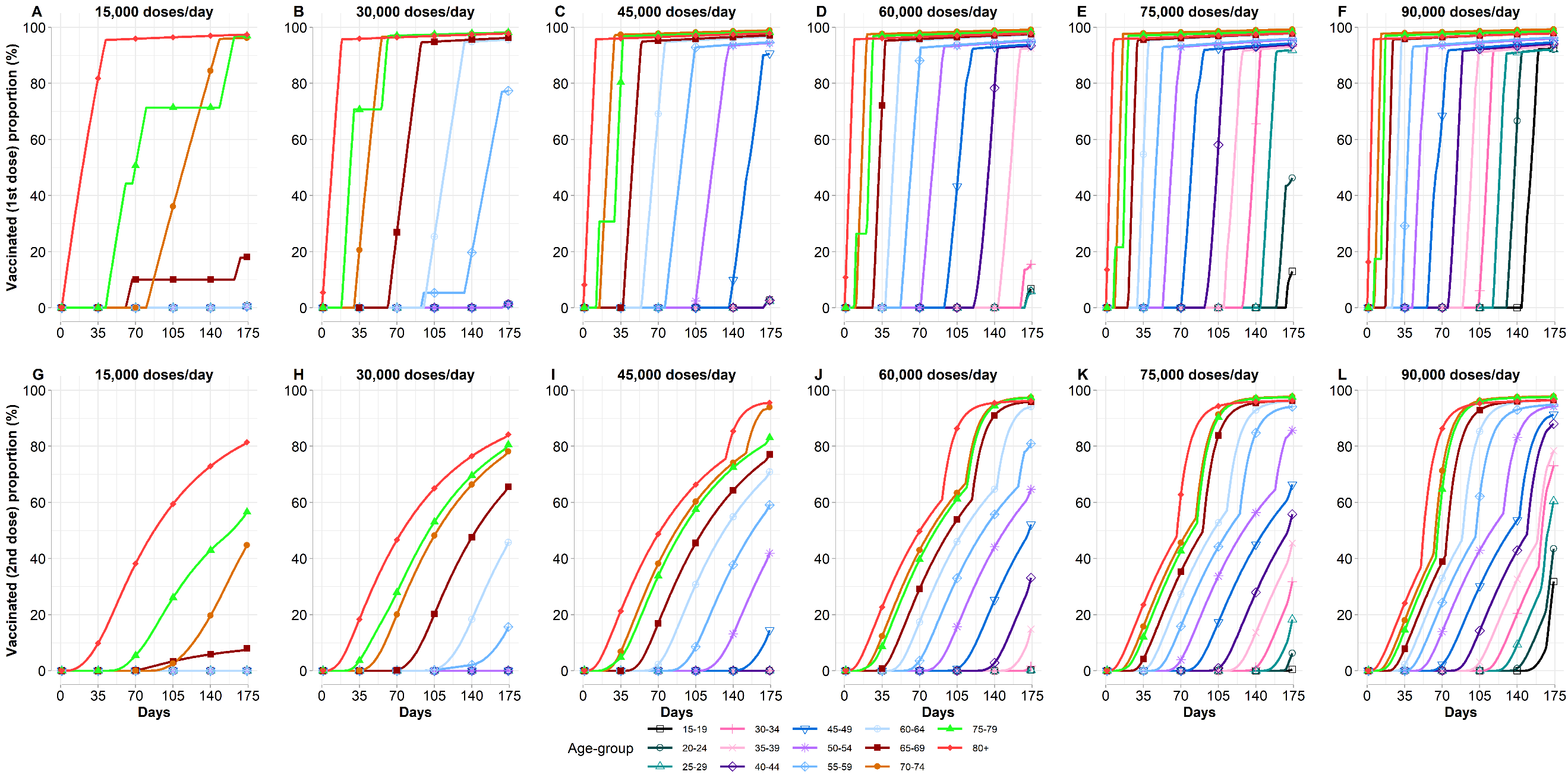


**Supplementary Figure 4.** Age-specific vaccinated proportions vary over time with respect to different levels of vaccine supply. **(A-F)** Vaccinated first dose proportions vary over time when the vaccine supply is 15,000, 30,000, 45,000, 60,000, 75,000, and 90,000 doses per day, respectively. **(G-L)** Vaccinated second dose proportions vary over time when the vaccine supply is 15,000, 30,000, 45,000, 60,000, 75,000, and 90,000 doses per day, respectively.


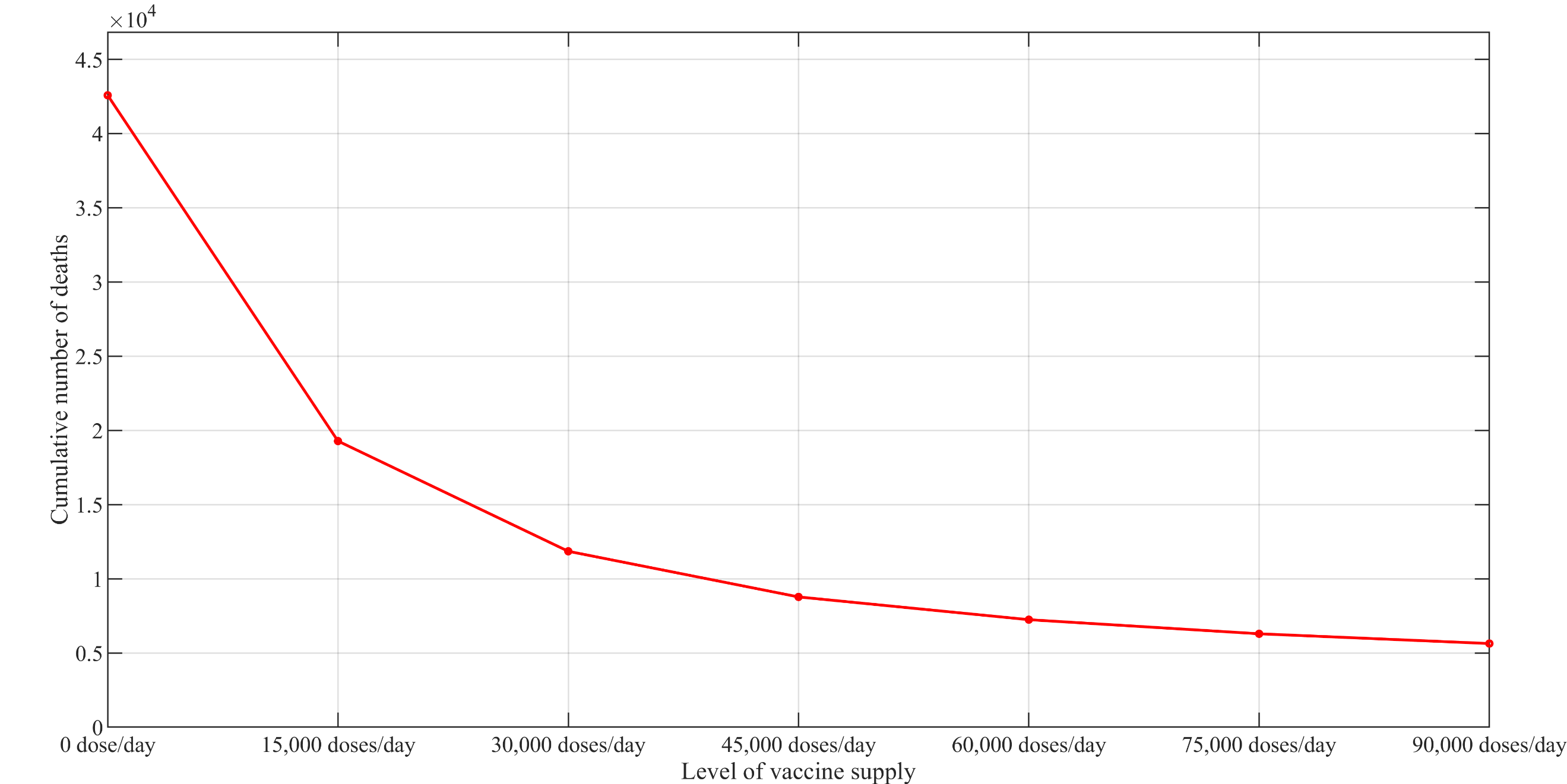


**Supplementary Figure 5.** Number of cumulative deaths with respect to different levels of vaccine supply.


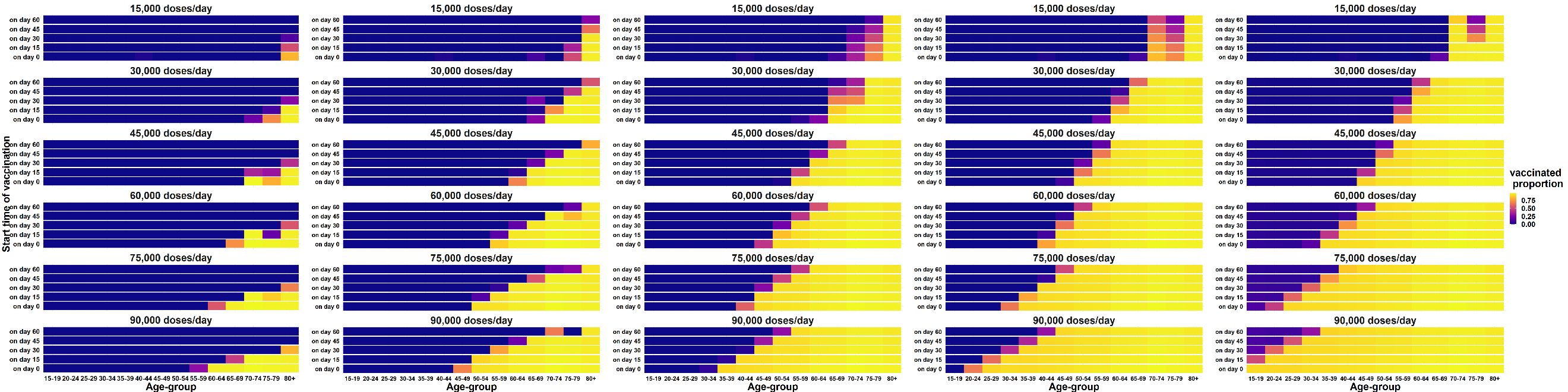


**Supplementary Figure 6.** Age-specific vaccinated (first dose) proportions with respect to different levels of vaccine supply and start time of vaccination.


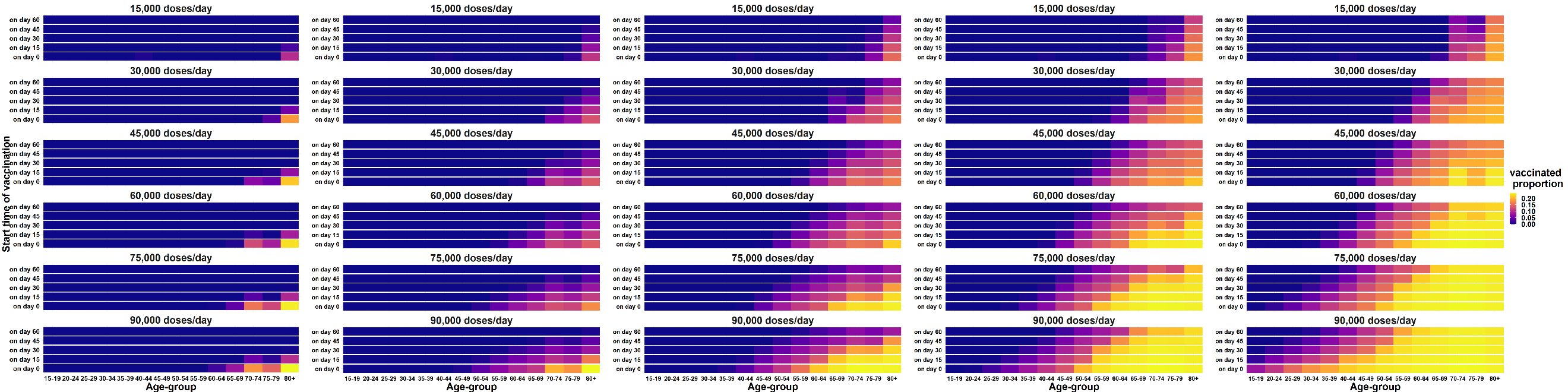


**Supplementary Figure 7.** Age-specific vaccinated (second dose) proportions with respect to different levels of vaccine supply and start time of vaccination.


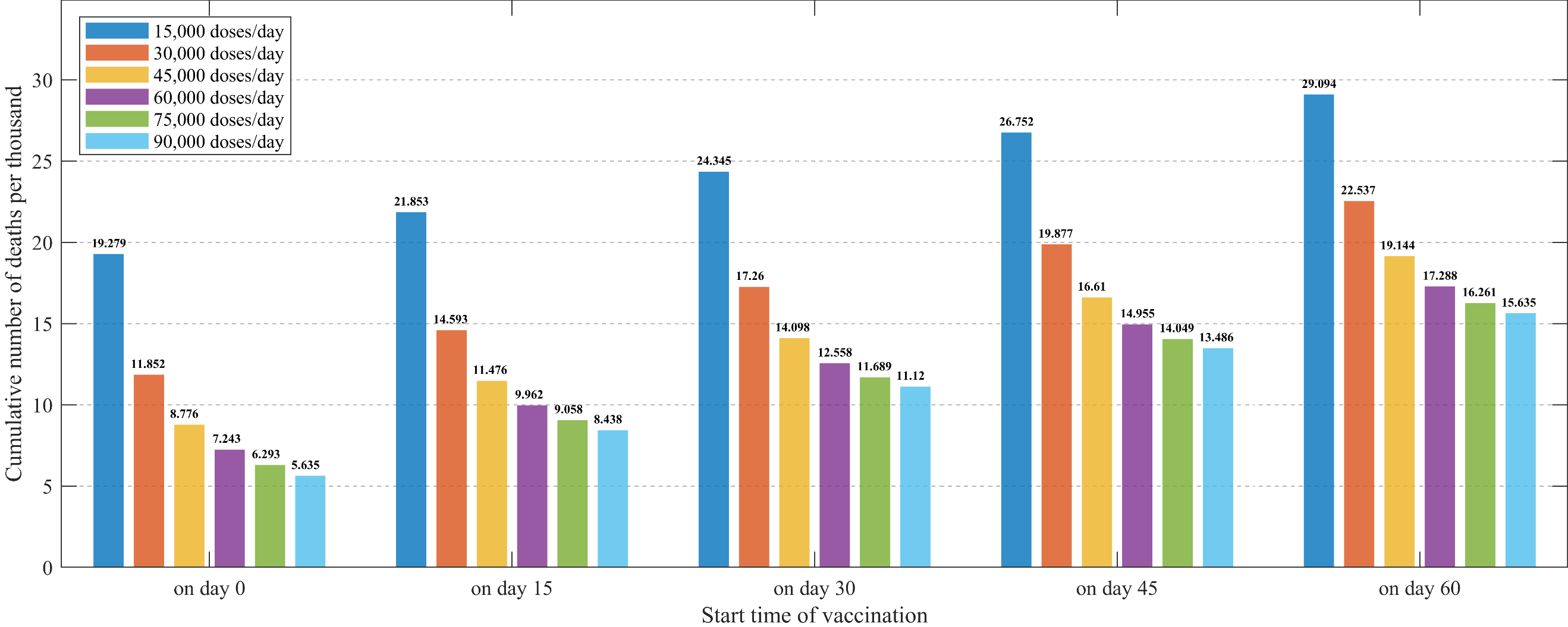


**Supplementary Figure 8.** Cumulative number of deaths with respect to different levels of vaccine supply and start time of vaccination.

**Appendix B. Additional tables cited in the main manuscript**

**Supplementary Table 1.** Summary of the disease transition parameter values and their sources.

| Parameters | Description | Value | Source |
| --- | --- | --- | --- |
| $\chi^{A}$ | Relative infectiousness of asymptomatic infectious individuals | 0.223 | ([1](#_ENREF_1), [2](#_ENREF_2)) |
| $\chi^{P}$ | Relative infectiousness of pre-symptomatic infectious individuals | 1 | ([1](#_ENREF_1), [2](#_ENREF_2)) |
| $\frac{1}{\alpha}$ | Mean duration of delay between receiving vaccine injection and the onset of dose-specific effectiveness | 10 | ([3](#_ENREF_3)) |
| $\frac{1}{\tau}$ | Mean duration of naturally acquired immunity | 240 | ([4-6](#_ENREF_4)) |
| $\frac{1}{\omega}$ | Mean latent period | 3.4 | ([1](#_ENREF_1), [2](#_ENREF_2)) |
|  | Mean duration of the pre-symptomatic infectious period | 1.7 | ([1](#_ENREF_1), [2](#_ENREF_2)) |
| $\frac{1}{\delta}$ | Mean duration of the mildly-symptomatic infectious period | 2.1 | ([1](#_ENREF_1), [2](#_ENREF_2)) |
| $\frac{1}{\lambda}$ | Mean duration of severe infection prior to hospitalization | 1.9 | ([1](#_ENREF_1), [2](#_ENREF_2)) |
| $\frac{1}{\eta^{AR}}$ | Mean recovery time of asymptomatic infectious individuals | 2.9 | ([1](#_ENREF_1), [2](#_ENREF_2)) |
| $\frac{1}{\eta^{ISMR}}$ | Mean recovery time of mildly-symptomatic infectious individuals | 4 | ([1](#_ENREF_1), [2](#_ENREF_2)) |
| $\frac{1}{\eta_{t}^{ICUR}}$ | Mean recovery time of ICU cases at time *t* | 15.6 on (and before) 2020-12-01;  15.0 on 2021-01-01;  13.1 on 2021-03-01;  9.3 on (and after) 2021-06-15 | ([2](#_ENREF_2)) |
| $\frac{1}{\eta_{t}^{HR}}$ | Mean recovery time of non-ICU cases at time *t* | 10.7 before 2020-12-01;  10.3 on 2021-01-01;  9.0 on 2021-03-01;  6.4 on (and after) 2021-06-15 | ([2](#_ENREF_2)) |
| $\frac{1}{\xi_{t}^{ICUD}}$ | Mean time of ICU cases who died at time *t* | 7 before 2020-12-01;  6.7on 2021-01-01;  5.9 on 2021-03-01;  4.2 on (and after) 2021-06-15 | ([2](#_ENREF_2)) |
| $\frac{1}{\xi_{t}^{HD}}$ | Mean time of non-ICU cases who die at time *t* | 10.3 before 2020-12-01;  9.9 on 2021-01-01;  8.6 on 2021-03-01;  6.1 on (and after) 2021-06-15 | ([2](#_ENREF_2)) |
| $\frac{1}{\varsigma_{_{interval}}^{U}}$ | Maximum time interval between the administration of the first dose and second dose | 84 | ([7](#_ENREF_7)) |
| $\frac{1}{\varsigma_{_{interval}}^{L}}$ | Minimum time interval between the administration of the first dose and second dose | 21 | ([7](#_ENREF_7)) |

**Supplementary Table 2.** Summary of age-stratified parameters for susceptibility, symptomatic, hospitalization, and death.

| Age-group | Relative susceptibility to infection $\rho_{j}$ |  | Proportion of exposed individuals who become pre-symptomatic $\sigma_{j}$ |  | Proportion of mildly-symptomatic infectious individuals who develops severe disease $\varepsilon_{j}$ |  | Proportion of hospitalized cases who required ICU $\psi_{j}$ |  | Proportion of ICU cases who died $\gamma_{j}$ |  | Proportion of non-ICU cases who died $\theta_{j}$ |
| --- | --- | --- | --- | --- | --- | --- | --- | --- | --- | --- | --- |
|  | ([8](#_ENREF_8)) |  | ([1](#_ENREF_1)) |  | ([2](#_ENREF_2)) |  | ([2](#_ENREF_2)) |  | ([1](#_ENREF_1)) |  | ([1](#_ENREF_1)) |
| 0-4 | 0.5995 |  | 0.2500 |  | 0.0335 |  | 0.0453 |  | 0.2171 |  | 0.0168 |
| 5-9 | 0.3546 |  | 0.2688 |  | 0.0009 |  | 0.0538 |  | 0.2202 |  | 0.0159 |
| 10-14 | 0.3987 |  | 0.3250 |  | 0.0052 |  | 0.0630 |  | 0.2241 |  | 0.0151 |
| 15-19 | 0.4292 |  | 0.4188 |  | 0.0077 |  | 0.0725 |  | 0.2302 |  | 0.0151 |
| 20-24 | 0.5991 |  | 0.5500 |  | 0.0224 |  | 0.0825 |  | 0.2387 |  | 0.0155 |
| 25-29 | 0.6864 |  | 0.5500 |  | 0.0344 |  | 0.0937 |  | 0.2526 |  | 0.0168 |
| 30-34 | 0.7005 |  | 0.5500 |  | 0.0361 |  | 0.1062 |  | 0.2718 |  | 0.0194 |
| 35-39 | 0.6759 |  | 0.5500 |  | 0.0387 |  | 0.1217 |  | 0.3003 |  | 0.0237 |
| 40-44 | 0.6944 |  | 0.5500 |  | 0.0430 |  | 0.1409 |  | 0.3434 |  | 0.0318 |
| 45-49 | 0.7498 |  | 0.5500 |  | 0.0636 |  | 0.1614 |  | 0.4004 |  | 0.0460 |
| 50-54 | 0.8735 |  | 0.5500 |  | 0.1187 |  | 0.1777 |  | 0.4651 |  | 0.0675 |
| 55-59 | 0.9714 |  | 0.5500 |  | 0.1703 |  | 0.1863 |  | 0.5429 |  | 0.1023 |
| 60-64 | 1.0218 |  | 0.5500 |  | 0.2124 |  | 0.1811 |  | 0.6206 |  | 0.1518 |
| 65-69 | 1.2635 |  | 0.5500 |  | 0.3560 |  | 0.1591 |  | 0.6922 |  | 0.2159 |
| 70-74 | 1.3442 |  | 0.5500 |  | 0.5487 |  | 0.1202 |  | 0.7461 |  | 0.2903 |
| 75-79 | 1.2771 |  | 0.5500 |  | 0.8600 |  | 0.0749 |  | 0.7700 |  | 0.3578 |
| 80+ | 1.4864 |  | 0.5500 |  | 0.7508 |  | 0.0199 |  | 0.7069 |  | 0.4300 |

**Supplementary Table 3.** The contact matrix between different age groups.

| Age-group | 0-4 | 5-9 | 10-14 | 15-19 | 20-24 | 25-29 | 30-34 | 35-39 | 40-44 | 45-49 | 50-54 | 55-59 | 60-64 | 65-69 | 70-74 | 75-79 | 80+ |
| --- | --- | --- | --- | --- | --- | --- | --- | --- | --- | --- | --- | --- | --- | --- | --- | --- | --- |
| 0-4 | 1.8531 | 0.7404 | 0.4283 | 0.2805 | 0.4233 | 0.6800 | 0.7268 | 0.8378 | 0.3732 | 0.2315 | 0.3077 | 0.2293 | 0.2279 | 0.1789 | 0.1268 | 0.0265 | 0.0195 |
| 5-9 | 0.8161 | 6.6020 | 1.2267 | 0.5564 | 0.4530 | 0.6712 | 0.9643 | 1.1518 | 0.9651 | 0.3355 | 0.3210 | 0.2077 | 0.3903 | 0.3066 | 0.1053 | 0.4166 | 0.3068 |
| 10-14 | 0.4613 | 1.1985 | 6.9872 | 1.3387 | 0.2419 | 0.3526 | 0.5242 | 0.9690 | 1.0830 | 0.6140 | 0.3371 | 0.3442 | 0.2402 | 0.2793 | 0.2696 | 0.2692 | 0.1982 |
| 15-19 | 0.2837 | 0.5105 | 1.2570 | 6.8817 | 1.2290 | 0.6851 | 0.4227 | 0.7555 | 0.8843 | 0.9298 | 0.5245 | 0.2963 | 0.2223 | 0.3610 | 0.4005 | 0.5663 | 0.4171 |
| 20-24 | 0.4862 | 0.4720 | 0.2580 | 1.3957 | 2.6581 | 1.3781 | 0.8071 | 0.6971 | 0.7885 | 0.9216 | 0.6549 | 0.5050 | 0.3797 | 0.3108 | 0.2547 | 0.3360 | 0.2474 |
| 25-29 | 0.8017 | 0.7179 | 0.3860 | 0.7987 | 1.4147 | 1.7905 | 1.0568 | 0.8283 | 0.8492 | 0.8877 | 0.8335 | 0.6910 | 0.5275 | 0.5091 | 0.2694 | 0.2318 | 0.1707 |
| 30-34 | 0.8272 | 0.9957 | 0.5539 | 0.4757 | 0.7998 | 1.0202 | 1.5842 | 1.1679 | 0.9179 | 0.7077 | 0.7151 | 0.6236 | 0.5896 | 0.3850 | 0.1412 | 0.5439 | 0.4006 |
| 35-39 | 0.9155 | 1.1418 | 0.9832 | 0.8164 | 0.6633 | 0.7678 | 1.1214 | 1.4033 | 1.2470 | 0.8840 | 0.6610 | 0.5680 | 0.6874 | 0.6357 | 0.1991 | 0.6560 | 0.4832 |
| 40-44 | 0.3805 | 0.8926 | 1.0252 | 0.8915 | 0.7000 | 0.7343 | 0.8223 | 1.1634 | 1.2929 | 1.1837 | 0.7189 | 0.5576 | 0.6887 | 0.5189 | 0.5573 | 0.8912 | 0.6564 |
| 45-49 | 0.2587 | 0.3401 | 0.6371 | 1.0275 | 0.8967 | 0.8415 | 0.6949 | 0.9040 | 1.2974 | 1.7892 | 0.7164 | 0.6241 | 0.5968 | 0.3635 | 0.4883 | 0.6212 | 0.4575 |
| 50-54 | 0.3747 | 0.3546 | 0.3812 | 0.6316 | 0.6944 | 0.8609 | 0.7652 | 0.7367 | 0.8587 | 0.7807 | 0.7306 | 0.9463 | 0.5419 | 0.4193 | 0.4156 | 1.4233 | 1.0483 |
| 55-59 | 0.2717 | 0.2233 | 0.3786 | 0.3471 | 0.5209 | 0.6944 | 0.6492 | 0.6158 | 0.6480 | 0.6617 | 0.9205 | 1.1843 | 0.8952 | 0.6183 | 0.3114 | 0.9126 | 0.6721 |
| 60-64 | 0.2288 | 0.3554 | 0.2238 | 0.2207 | 0.3319 | 0.4491 | 0.5199 | 0.6313 | 0.6780 | 0.5361 | 0.4467 | 0.7584 | 0.6594 | 0.6373 | 0.3903 | 1.0820 | 0.7968 |
| 65-69 | 0.1607 | 0.2499 | 0.2330 | 0.3207 | 0.2431 | 0.3879 | 0.3039 | 0.5226 | 0.4572 | 0.2922 | 0.3093 | 0.4688 | 0.5704 | 0.7144 | 0.5884 | 0.3071 | 0.2261 |
| 70-74 | 0.1149 | 0.0865 | 0.2268 | 0.3588 | 0.2009 | 0.2070 | 0.1124 | 0.1651 | 0.4953 | 0.3959 | 0.3092 | 0.2381 | 0.3523 | 0.5934 | 0.6188 | 0.8328 | 0.6134 |
| 75-79 | 0.0075 | 0.1070 | 0.0707 | 0.1585 | 0.0828 | 0.0556 | 0.1353 | 0.1699 | 0.2474 | 0.1573 | 0.3308 | 0.2180 | 0.3051 | 0.0968 | 0.2602 | 0.9599 | 0.7069 |
| 80+ | 0.0101 | 0.1446 | 0.0956 | 0.2142 | 0.1119 | 0.0752 | 0.1828 | 0.2296 | 0.3344 | 0.2126 | 0.4470 | 0.2946 | 0.4123 | 0.1307 | 0.3516 | 1.2971 | 0.9553 |

**Supplementary Table 4.** Summary of age-stratified parameters for initial conditions and population size.

| Age-group | Proportion initially infected |  | Proportion initially recovered |  | Proportion initially deceased |  | Proportion initially cases in the general ward |  | Proportion initially cases in the ICU |  | Population size |
| --- | --- | --- | --- | --- | --- | --- | --- | --- | --- | --- | --- |
|  | ([9](#_ENREF_9)) |  | ([3](#_ENREF_3)) |  | ([9](#_ENREF_9)) |  | ([3](#_ENREF_3)) |  | ([3](#_ENREF_3)) |  | ([10](#_ENREF_10)) |
| 0-4 | 0.0237 |  | 0.1410 |  | 0.0002 |  | 0.0029 |  | 0.0019 |  | 612891 |
| 5-9 | 0.0631 |  | 0.1410 |  | 0.0000 |  | 0.0029 |  | 0.0019 |  | 675602 |
| 10-14 | 0.0894 |  | 0.0767 |  | 0.0002 |  | 0.0054 |  | 0.0035 |  | 660108 |
| 15-19 | 0.0759 |  | 0.0767 |  | 0.0004 |  | 0.0054 |  | 0.0035 |  | 619822 |
| 20-24 | 0.0801 |  | 0.0873 |  | 0.0009 |  | 0.0234 |  | 0.0151 |  | 703932 |
| 25-29 | 0.0822 |  | 0.0873 |  | 0.0013 |  | 0.0234 |  | 0.0151 |  | 722600 |
| 30-34 | 0.0865 |  | 0.0663 |  | 0.0025 |  | 0.0303 |  | 0.0195 |  | 697565 |
| 35-39 | 0.0831 |  | 0.0663 |  | 0.0052 |  | 0.0303 |  | 0.0195 |  | 669776 |
| 40-44 | 0.078 |  | 0.0513 |  | 0.0067 |  | 0.0456 |  | 0.0489 |  | 624878 |
| 45-49 | 0.0708 |  | 0.0513 |  | 0.0127 |  | 0.0456 |  | 0.0489 |  | 684924 |
| 50-54 | 0.0699 |  | 0.0405 |  | 0.0195 |  | 0.0725 |  | 0.1116 |  | 746423 |
| 55-59 | 0.0585 |  | 0.0405 |  | 0.0303 |  | 0.0725 |  | 0.1116 |  | 726145 |
| 60-64 | 0.0421 |  | 0.0199 |  | 0.0367 |  | 0.0774 |  | 0.1446 |  | 615188 |
| 65-69 | 0.0274 |  | 0.0199 |  | 0.0739 |  | 0.0774 |  | 0.1446 |  | 550582 |
| 70-74 | 0.0222 |  | 0.0095 |  | 0.0982 |  | 0.1004 |  | 0.1224 |  | 555286 |
| 75-79 | 0.0168 |  | 0.0095 |  | 0.1383 |  | 0.1004 |  | 0.1224 |  | 409037 |
| 80+ | 0.0301 |  | 0.0152 |  | 0.5731 |  | 0.2847 |  | 0.0655 |  | 552753 |

**Supplementary Table 5.** Summary of vaccine efficacy against infection, symptomatic disease, and severe disease.

| Vaccine efficacy against | Vaccinated with the first dose (protection has yet to be realized) | Vaccinated with the first dose (protected by the vaccine effect of the first dose) | Vaccinated with the second dose (not improve the protection efficiency yet) | Vaccinated with the second dose (protected by the full vaccine effect of the two doses) | Source |
| --- | --- | --- | --- | --- | --- |
| Infection | 0.00% | 34.00% | 34.00% | 71.00% | ([11](#_ENREF_11)) |
| Symptomatic disease | 0.00% | 39.70% | 39.70% | 83.55% | ([12](#_ENREF_12)) |
| Severe disease | 0.00% | 80.00% | 80.00% | 95.00% | ([13](#_ENREF_13)) |

**Appendix C. Additional case study results**

In this section, we provide additional case study results in two distinct National Health Service (NHS) regions: East of England (EE) and North East and Yorkshire (NEY). All the case study data are primarily from a series of references and public sources, and the source of the data has been described in the main text. [**Supplementary Figure 9**](#Supplementary_Fig_9) and [**Supplementary Figure 10**](#Supplementary_Fig_10) present an intuitive comparison between the officially reported pandemic data (red circles, solid line) and the prediction results of the model (blue asterisks, dashed line) in the East of England and North East and Yorkshire, respectively. Both figures demonstrate that the proposed model provides an excellent fit for the cumulative number of deaths, hospital admissions, and hospital bed occupancy, other than a light overestimation for the hospital bed occupancy in the North East and Yorkshire. Moreover, as in the main text, we further evaluated the performance of the model using three common metrics, including the mean absolute percentage error (MAPE), the normalized root mean squared error (*n*RMSE), and the explained variance between the officially reported pandemic data and the prediction results of the model, as shown in [**Supplementary Table 6**](#Supplementary_Tab_6). The results indicated that the MAPE and *n*RMSR values are pretty low and explained variance values close to 1. This further suggests that the model presented in the paper is reliable.


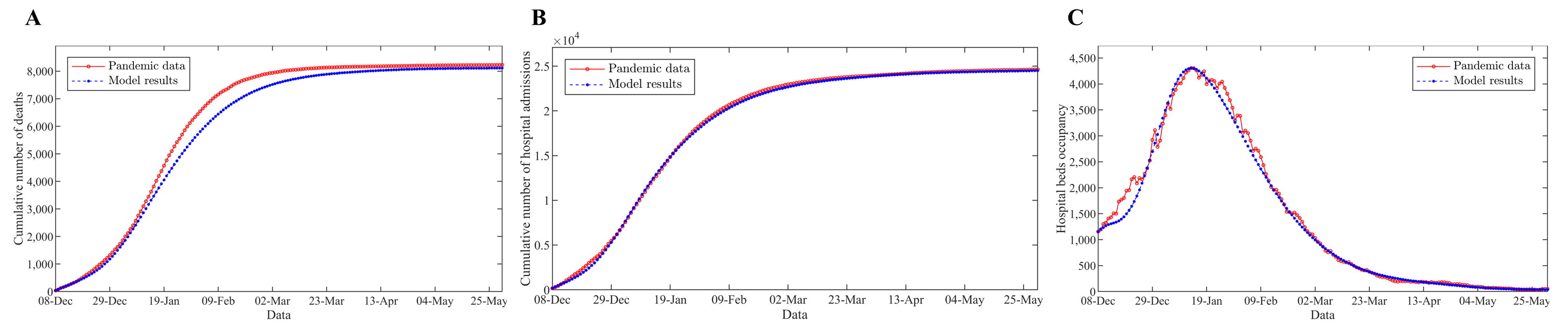


**Supplementary Figure 9.** Comparison of the officially reported pandemic data and the model results in the East of England. **(A)** Cumulative number of deaths. **(B)** Cumulative number of hospital admissions. **(C)** Hospital beds occupancy.


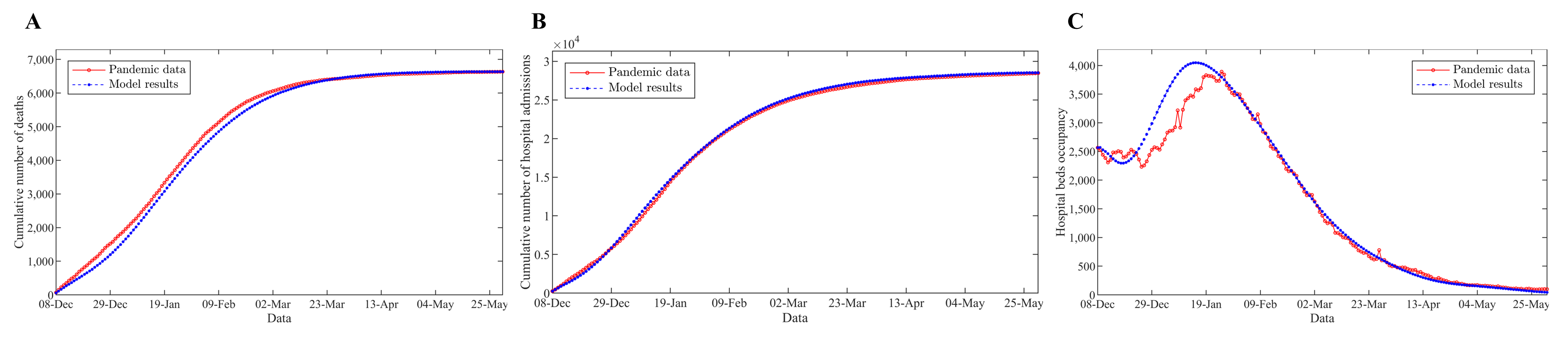


**Supplementary Figure 10.** Comparison of the officially reported pandemic data and the model results in the North East and Yorkshire. **(A)** Cumulative number of deaths. **(B)** Cumulative number of hospital admissions. **(C)** Hospital beds occupancy.

**Supplementary Table 6.** Statistical analysis to compare the officially reported pandemic data and the model results in the East of England and North East and Yorkshire.

| Region | Data | Metric | | |
| --- | --- | --- | --- | --- |
|  |  | Mean absolute percentage error (%) | Normalized root mean squared error | Explained variance (%) |
| East of England | Cumulative number of deaths | 5.46 | 0.0578 | 99.36 |
|  | Cumulative number of hospital admissions | 2.16 | 0.0112 | 99.96 |
|  | Hospital beds occupancy | 8.04 | 0.0922 | 99.25 |
| North East and Yorkshire | Cumulative number of deaths | 6.02 | 0.0359 | 99.60 |
|  | Cumulative number of hospital admissions | 2.48 | 0.0139 | 99.94 |
|  | Hospital beds occupancy | 10.78 | 0.1542 | 96.78 |

In addition, we also show that optimal allocation strategy by solving the multi-period two-dose vaccine allocation model with an application to the COVID-19 vaccination campaign in the East of England and North East and Yorkshire. [**Supplementary Figure 11**](#Supplementary_Fig_11) and [**Supplementary Figure 12**](#Supplementary_Fig_12) present the number of the first and second doses of vaccine were allocated per day and the vaccinated proportion varies over time, respectively. [**Supplementary Figure 13**](#Supplementary_Fig_13) and [**Supplementary Figure 14**](#Supplementary_Fig_14) show the vaccine coverages vary over time per age group in the East of England and North East and Yorkshire, respectively. We found that all of the main conclusions of the main text are kept the same.


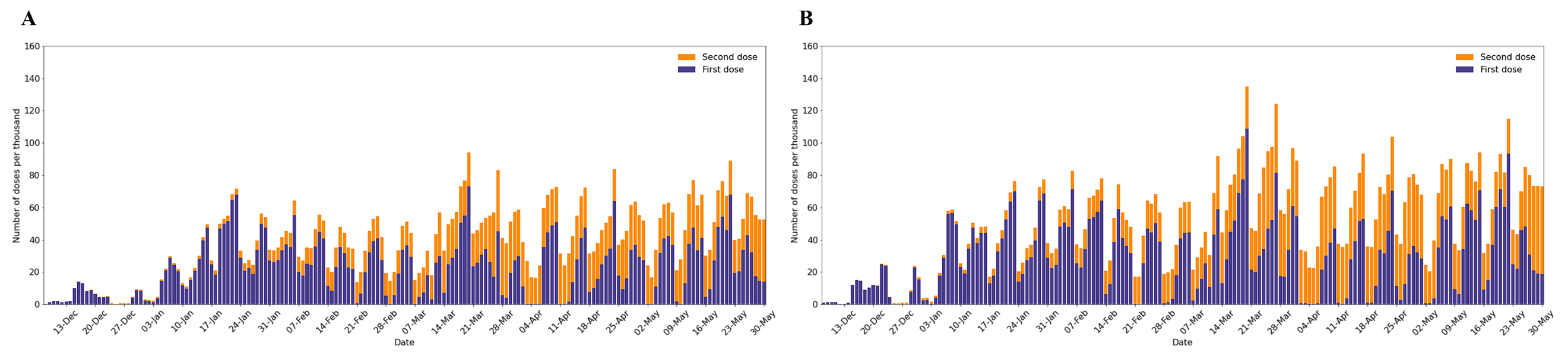


**Supplementary Figure 11.** Number of daily allocated vaccine doses. **(A)** East of England. **(B)** North East and Yorkshire.


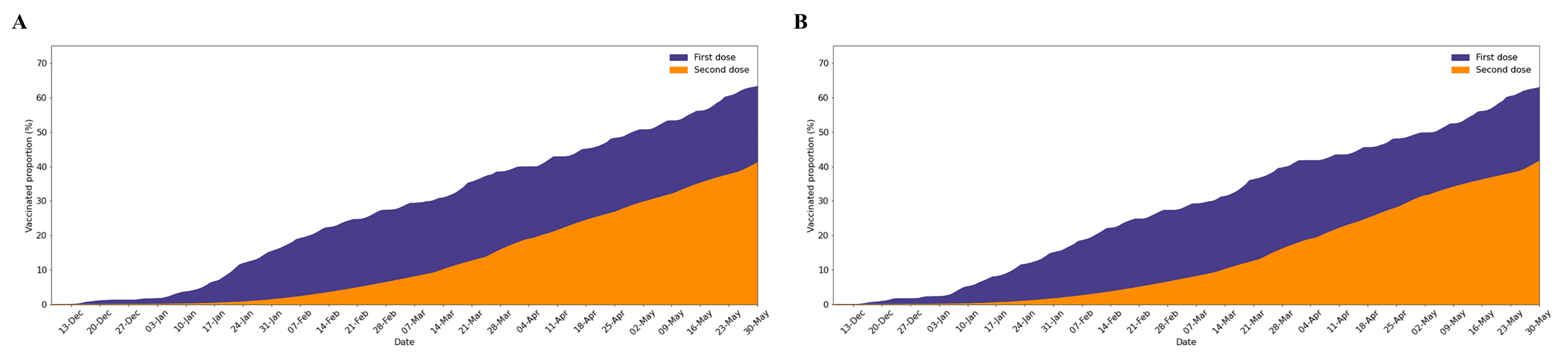


**Supplementary Figure 12.** Vaccinated proportion varies over time. **(A)** East of England. **(B)** North East and Yorkshire.


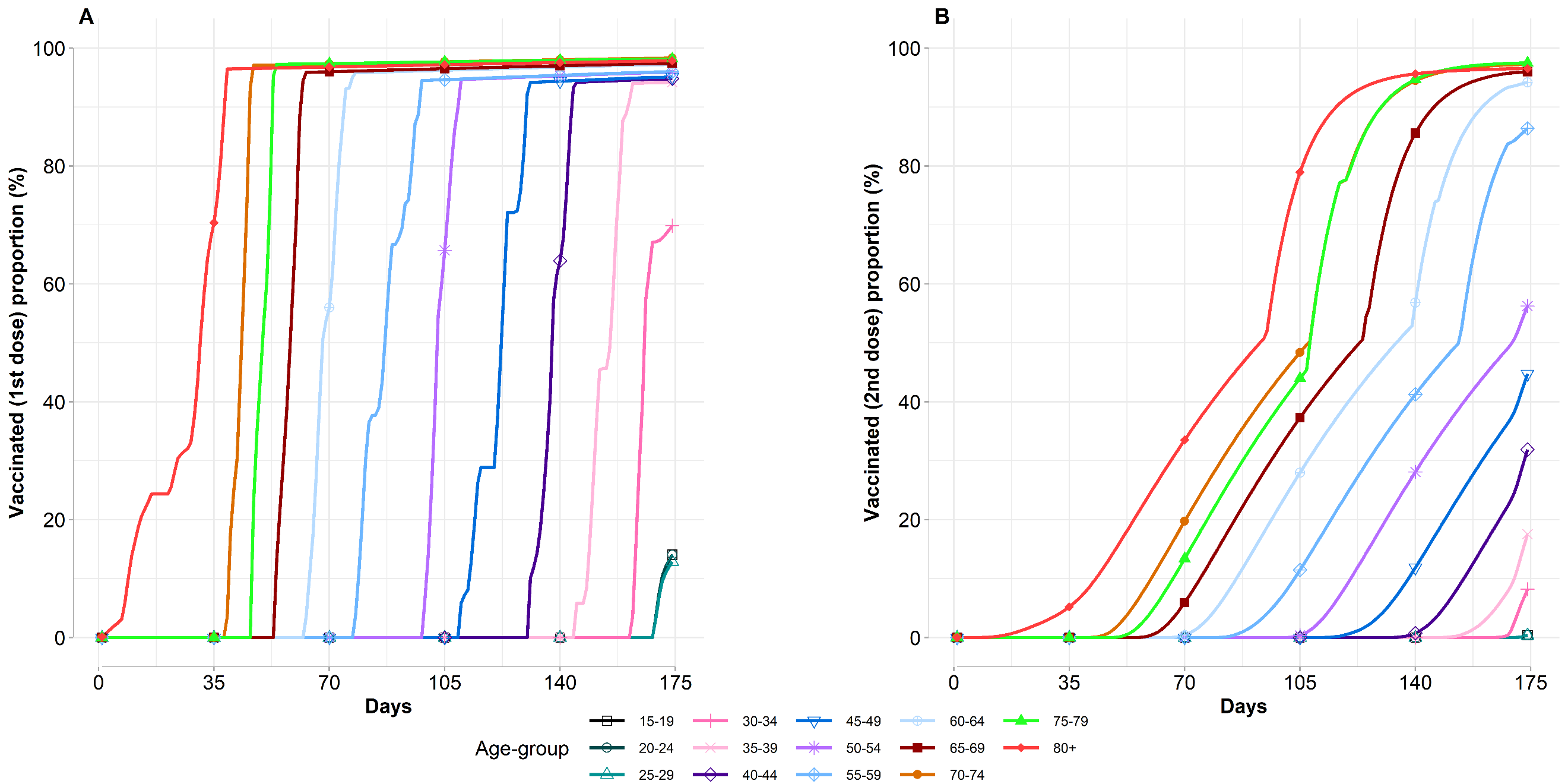


**Supplementary Figure 13.** Age-specific vaccinated proportions vary over time in the East of England. **(A)** Vaccinated first dose proportion. **(B)** Vaccinated second dose proportion.


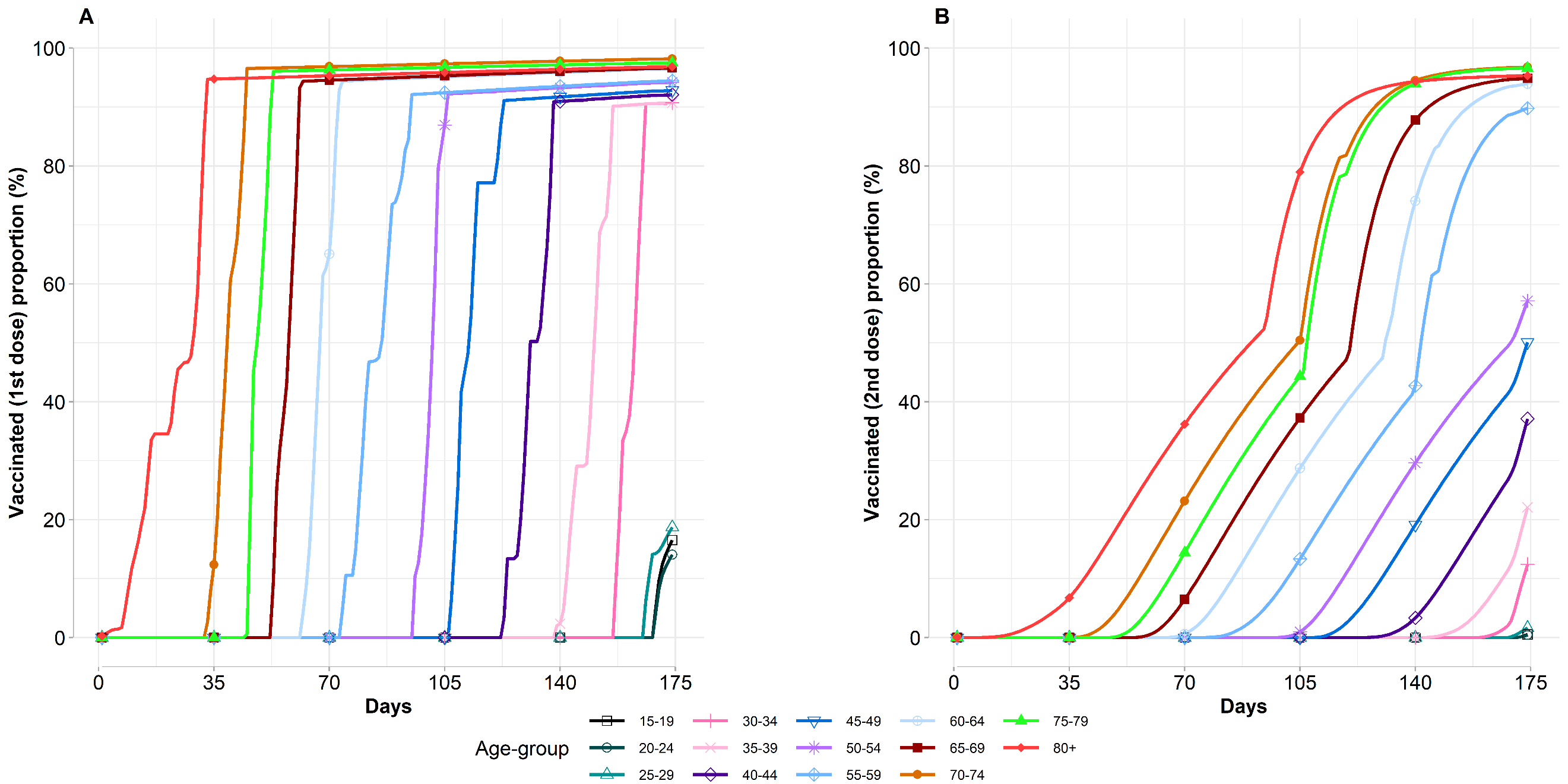


**Supplementary Figure 14.** Age-specific vaccinated proportions vary over time in the North East and Yorkshire. **(A)** Vaccinated first dose proportion. **(B)** Vaccinated second dose proportion.

**Appendix D. Vaccine hesitancy**

In the main text, we considered that all vaccine-eligible individuals were willing to be vaccinated. This is a strong assumption. Therefore, in this section, we introduce a parameter *μ* to represent the rate of vaccine hesitance, and the proposed model is extended by adding constraint (S1) in order to ensure that the number of vaccines allocated to individuals in age group *j* who administer the first dose is not more than the number of unvaccinated susceptible individuals in age group *j* who want to receive the vaccine. We performed a sensitivity analysis by running the model using six different values of the fraction of the population refusing the vaccine: 0%, 10%, 20%, 30%, 40%, and 50%. **[Supplementary Figure 15](#Supplementary_Fig_15)** summarizes the optimal vaccine allocation strategies for various rates of vaccine hesitance. Similar to the results given in the main text, vaccine resources should be prioritized for older populations to mitigate the impacts of the pandemic in terms of the number of deaths, whatever the vaccine hesitancy rate was. In [**Supplementary Figure 16**](#Supplementary_Fig_16), we show the cumulative number of deaths with respect to different rates of vaccine hesitance, respectively. As expected, we find an increase in the number of deaths as the rate of vaccine hesitance rise.

$\sum_{t\in T} x_{j,t}\leq N_{j}(1-\mu),\text{ }\forall j\in J$ (S1)


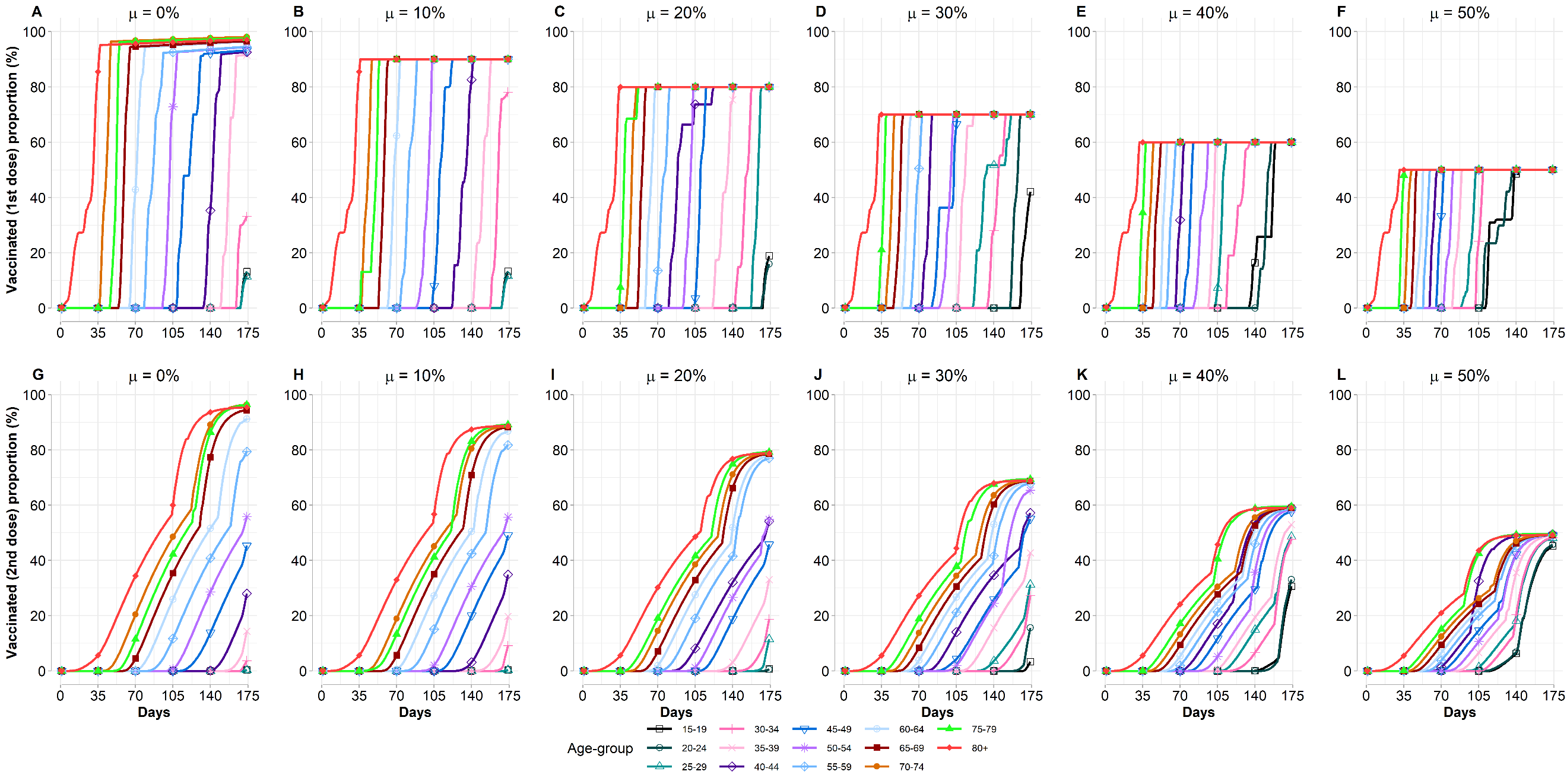


**Supplementary Figure 15.** Age-specific vaccinated proportions vary over time with respect to different rates of vaccine hesitance. **(A-F)** Vaccinated first dose proportions vary over time when the rate of vaccine hesitance is 0%, 10%, 20%, 30%, 40%, and 50%, respectively. **(G-L)** Vaccinated second dose proportions vary over time when the rate of vaccine hesitance is 0%, 10%, 20%, 30%, 40%, and 50%, respectively.


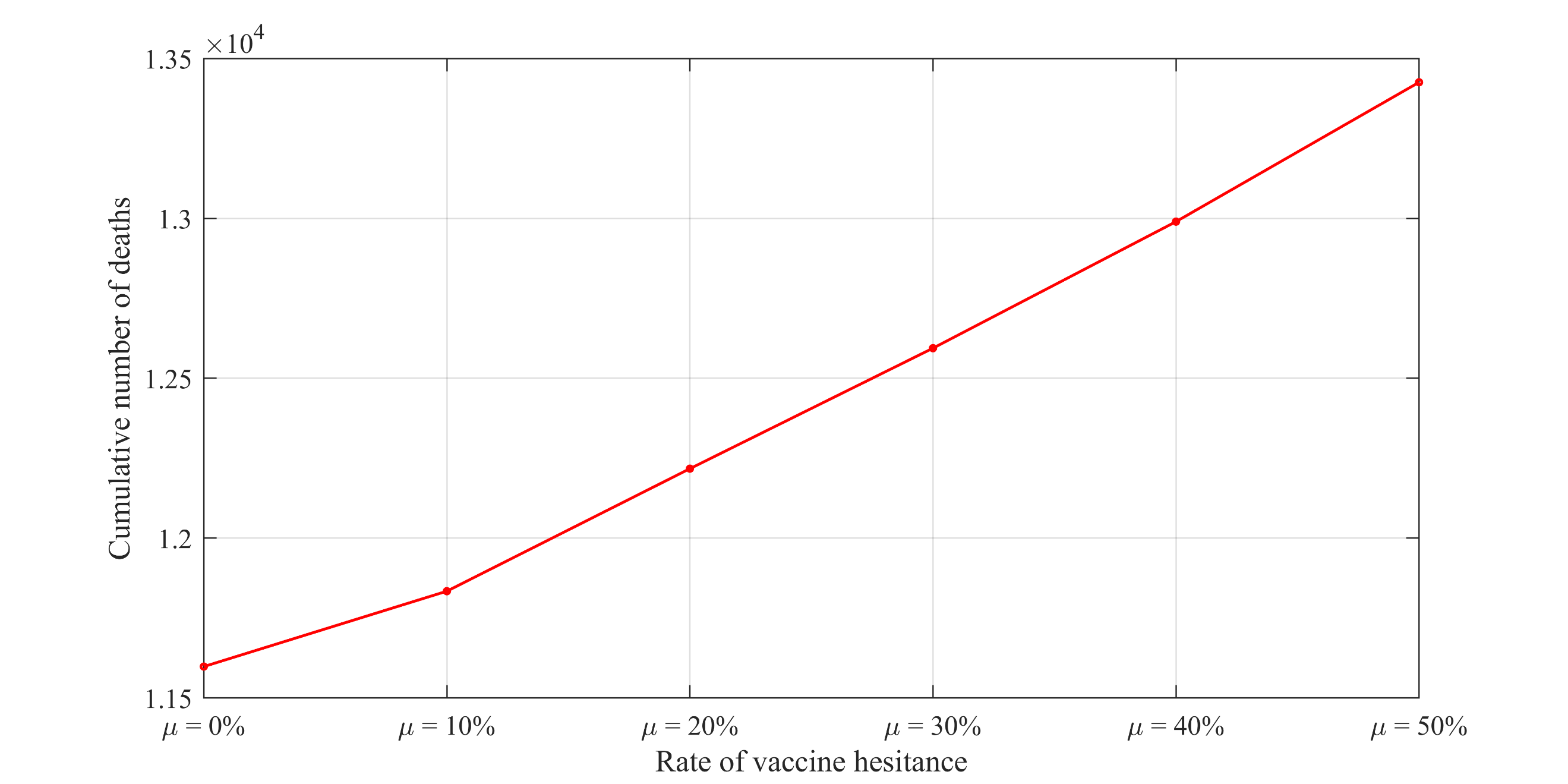


**Supplementary Figure 16.** Cumulative number of deaths with respect to different rates of vaccine hesitance.

# REFERENCES

1. Knock ES, Whittles LK, Lees JA, Perez-Guzman PN, Verity R, FitzJohn RG, et al. Key Epidemiological Drivers and Impact of Interventions in the 2020 Sars-Cov-2 Epidemic in England. *Sci Transl Med*. (2021) 13(602):eabg4262. doi: doi:10.1126/scitranslmed.abg4262.

2. Sonabend R, Whittles LK, Imai N, Perez-Guzman PN, Knock ES, Rawson T, et al. Non-Pharmaceutical Interventions, Vaccination, and the Sars-Cov-2 Delta Variant in England: A Mathematical Modelling Study. *Lancet*. (2021) 398(10313):1825-35. doi: 10.1016/S0140-6736(21)02276-5.

3. Molla J, Ponce de Leon Chavez A, Hiraoka T, Ala-Nissila T, Kivela M, Leskela L. Adaptive and Optimized Covid-19 Vaccination Strategies across Geographical Regions and Age Groups. *PLoS Comput Biol*. (2022) 18(4):e1009974. doi: 10.1371/journal.pcbi.1009974.

4. World Health Organization. Covid-19 Natural Immunity: Scientific Brief, 10 May 2021. (2021). Available online at: <https://apps.who.int/iris/handle/10665/341241.html>. (accessed June 15, 2022).

5. Dan JM, Mateus J, Kato Y, Hastie KM, Yu ED, Faliti CE, et al. Immunological Memory to Sars-Cov-2 Assessed for up to 8 Months after Infection. *Science*. (2021) 371(6529). doi: 10.1126/science.abf4063.

6. Kojima N, Klausner JD. Protective Immunity after Recovery from Sars-Cov-2 Infection. *Lancet Infect Dis*. (2022) 22(1):12-4. doi: 10.1016/S1473-3099(21)00676-9.

7. Souto Ferreira L, Canton O, da Silva RLP, Poloni S, Sudbrack V, Borges ME, et al. Assessing the Best Time Interval between Doses in a Two-Dose Vaccination Regimen to Reduce the Number of Deaths in an Ongoing Epidemic of Sars-Cov-2. *PLoS Comput Biol*. (2022) 18(3):e1009978. doi: 10.1371/journal.pcbi.1009978.

8. Moore S, Hill EM, Tildesley MJ, Dyson L, Keeling MJ. Vaccination and Non-Pharmaceutical Interventions for Covid-19: A Mathematical Modelling Study. *Lancet Infect Dis*. (2021) 21(6):793-802. doi: 10.1016/S1473-3099(21)00143-2.

9. GOV. UK. Coronavirus (Covid-19) in the Uk. (2022). Available online at: <https://coronavirus.data.gov.uk.html>. (accessed June 9, 2022).

10. Office for National Statistics. Estimates of the Population for the Uk, England and Wales, Scotland and Northern Ireland. (2021). Available online at: <https://www.ons.gov.uk/peoplepopulationandcommunity/populationandmigration/populationestimates/datasets/populatione-stimatesforukenglandandwalesscotlandandnorthernireland.html> (accessed June 6, 2022).

11. Keeling MJ, Moore SE. An Assessment of the Vaccination of School-Aged Children in England against Sars-Cov-2. *BMC medicine*. (2022) 20(1):1-14. doi: 10.1186/s12916-022-02379-0.

12. Lopez Bernal J, Andrews N, Gower C, Gallagher E, Simmons R, Thelwall S, et al. Effectiveness of Covid-19 Vaccines against the B.1.617.2 (Delta) Variant. *N Engl J Med*. (2021) 385(7):585-94. doi: 10.1056/NEJMoa2108891.

13. UK Health Security Agency. Covid-19 Vaccine Weekly Surveillance Reports. (2021). Available online at: <https://www.gov.uk/government/-publications/covid-19-vaccine-weekly-surveillance-reports-full-publication-update-history.html>. (accessed April 6, 2022).
